# Infiltrated Photonic Crystal Fibers for Sensing Applications

**DOI:** 10.3390/s18124263

**Published:** 2018-12-04

**Authors:** José Francisco Algorri, Dimitrios C. Zografopoulos, Alberto Tapetado, David Poudereux, José Manuel Sánchez-Pena

**Affiliations:** 1 GDAF-UC3M, Displays and Photonics Applications Group, Electronic Technology Department, Carlos III University of Madrid, Leganés, 28911 Madrid, Spain; atapetad@ing.uc3m.es (A.T.); jmpena@ing.uc3m.es (J.M.S.-P.); 2 Consiglio Nazionale delle Ricerche, Istituto per la Microelettronica e Microsistemi, 00133 Rome, Italy; dimitrios.zografopoulos@artov.imm.cnr.it; 3 Alter Technoology TÜV Nord S.A.U. C/La Majada 3, 28760 Tres Cantos, Madrid, Spain; dpoudere@gmail.com

**Keywords:** photonic crystal fibers, optical fiber sensors, optofluidics, plasmonic sensors, liquid crystals

## Abstract

Photonic crystal fibers (PCFs) are a special class of optical fibers with a periodic arrangement of microstructured holes located in the fiber’s cladding. Light confinement is achieved by means of either index-guiding, or the photonic bandgap effect in a low-index core. Ever since PCFs were first demonstrated in 1995, their special characteristics, such as potentially high birefringence, very small or high nonlinearity, low propagation losses, and controllable dispersion parameters, have rendered them unique for many applications, such as sensors, high-power pulse transmission, and biomedical studies. When the holes of PCFs are filled with solids, liquids or gases, unprecedented opportunities for applications emerge. These include, but are not limited in, supercontinuum generation, propulsion of atoms through a hollow fiber core, fiber-loaded Bose–Einstein condensates, as well as enhanced sensing and measurement devices. For this reason, infiltrated PCF have been the focus of intensive research in recent years. In this review, the fundamentals and fabrication of PCF infiltrated with different materials are discussed. In addition, potential applications of infiltrated PCF sensors are reviewed, identifying the challenges and limitations to scale up and commercialize this novel technology.

## 1. Introduction

Over the past several decades, the advances in optical fiber technology have undoubtedly improved and reshaped the field of telecommunication technologies [1,2,3,4]. In parallel, breakthroughs in optical fiber manufacturing and photonics science have driven the use of fiber-optic components in applications beyond their traditional market. A sector that has significantly benefited from these advances is that of optical fiber sensors [5].

Optical fibers offer the potential for small and lightweight sensors, which are easily multiplexed and exhibit immunity to electromagnetic fields [6]. Nevertheless, despite these advantages, fiber-optic sensing technology had not proven to be an advantageous alternative to traditional sensors for a long time. This lack of success was due to the high cost and complexity of the interrogation techniques [7,8,9,10,11,12], which largely limited the use of fiber-optic sensors as low-cost components in sensing applications.

This trend changed with the ability to engineer the refractive index profile of optical fibers. In 1991, Phillip Russell conceived the idea of a special optical fiber combining the properties of photonic crystals (PC) with conventional optical fibers [13]. In 1995, his group suggested the possibility to guide light by a PC arrangement of microcapillaries running along the fiber axis [14], thus paving the way for a new kind of microstructured fibers called photonic crystal fibers (PCF). PCF broadly extended the operating field of optical fibers thanks to the high versatility in engineering their optical parameters and the possibility to infiltrate them with gaseous, liquid, or even solid materials.

**Figure 1:**
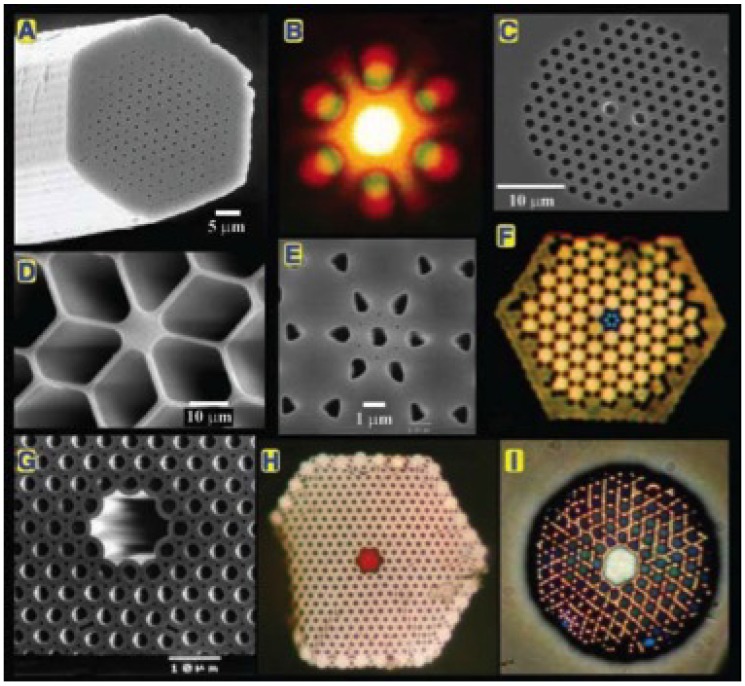
An assortment of optical (OM) and scanning electron (SEM) micrographs of PCF structures. (**A**) SEM of an endlessly single-mode solid core PCF; (**B**) far-field optical pattern produced by the fiber in (**A**) when excited by red and green laser light; (**C**) SEM of a recent birefringent PCF; (**D**) SEM of a small (800 nm) core PCF with ultrahigh nonlinearity and a zero chromatic dispersion at a wavelength of 560 nm; (**E**) SEM of the first photonic bandgap PCF, its core formed by an additional air hole in a graphite lattice of air holes; (**F**) near-field OM of the six-leaved blue mode that appears when the fiber shown in (**E**) is excited by white light; (**G**) SEM of a hollow-core photonic bandgap fiber; (**H**) near-field OM of a red mode in hollow-core PCF (white light is launched into the core); (**I**) OM of a hollow-core PCF with a Kagome cladding lattice, guiding white light, reprinted with permission from [13].

Photonic crystals are periodic structures that affect the motion of photons in a similar way that crystalline lattices affect ions in solids. The periodic structure of PC can extend in one, two or three dimensions. The main PC property is the appearance of photonic bandgaps (PBG), namely forbidden ranges of wavelengths, equivalent to the electronic bandgaps of semiconductors, in which light cannot propagate through the structure and is therefore reflected. If a low-index or even hollow-core (HC) fiber is surrounded by a 2D photonic crystal, then the bandgap-guiding (BG) mechanism is able to efficiently guide the light in the fiber core. Since the PBG wavelengths cannot cross through the periodic cladding, light is forced to travel through the central air channel as, for instance, in the PCF shown in Figure 1G.

The first performing PCF was manufactured in November 1995. It featured a hexagonal close-packed array of small air channels and was free of major imperfections or defects [14]. Although it turned out that the PCF was a guiding light via the index-guiding (IG) mechanism, from the structural point of view, it was the first fiber-photonic equivalent of a pure dopant- and defect-free semiconductor crystal. Most importantly, it demonstrated that the periodicity of the microcapillaries can be intentionally disrupted by introducing defects, which in the following years led to the design and fabrication of a very broad range of different PCF, as shown in Figure 1.

That first index-guiding PCF consisted of an array of approximately 300 nm air holes spaced by 2.3 µm around a central solid core [15]. It had the property to be single-mode no matter how short the light wavelength. This can be understood by viewing the array of holes in the cladding as a modal filter. The effective refractive index of the composite glass-air cladding is higher for shorter wavelengths, reducing the index difference between the cladding and the index-guiding solid PCF core and thus limiting the number of guided modes. A proper selection of the PCF geometry guarantees that only the fundamental mode is guided. More detailed studies show that this occurs for d/Λ<0.4, where *d* is the capillary diameter and Λ the pitch of the cladding’s hexagonal lattice [16]. By exploiting this property, very large mode-area fibers were designed offering big improvements in high-power delivery, amplifiers, and lasers [17]. Additionally, capillaries with different sizes and shapes can be introduced in the cladding so as to deliberately break the PCF symmetry and induce very high values of birefringence, unachievable by standard optical fibers [18].

**Figure 2:**
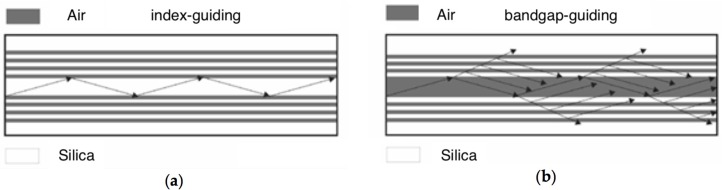
Guiding mechanisms in a PCF: (**a**) index-guiding. (**b**) bandgap-guiding through the photonic bandgap effect.

Overall, index-guiding PCF are similar to conventional fibers in the sense that their core has a larger refractive index than that of the cladding. Although standard PCF are made entirely of silica, the composite air-hole cladding has a lower effective refractive index than that of the solid core (Figure 2a), which shows strong wavelength-dispersion. The modes of all single-material, index-guiding PCF are leaky modes because the core refractive index is the same as that beyond the finite holey cladding. When the latter is composed of a small number of capillary rings, significant confinement losses may arise [19], also known as geometric losses [20]. As the guided modes are essentially leaky, the decay of the fields is exponential along the direction of propagation. Furthermore, the continuum of radiative modes still exists and the range of propagation constants associated with radiative modes is not disjoint from those of the leaky modes. This complicates the task of identifying the leaky guided modes, since a leaky mode can be “lost” within a continuum of radiative solutions to the wave equations. As a general remark, the complex structure of PCF renders difficult their mathematical study, and the well-known field of standard optical fibers is of little help, such that in most cases Maxwell’s equations must be numerically solved [21,22].

When a PCF has a hollow core or, generelly, a lower refractive index in the core than in the cladding, the light is guided by the photonic bandgap effect. The cladding is built by a periodic microstructure that forms a two-dimensional photonic crystal in the transverse plane, whose dielectric properties are characterized by photonic bandgaps. The periodic structure inside the PCF is locally broken, by creating the lower-index core usually by omitting one or more glass capillaries during fabrication. By proper geometrical design, it is possible to achieve light guidance in the hollow core of the PCF at those wavelengths for which transverse leakage through the cladding is forbidden by corresponding bandgaps (Figure 2b). As a result, light can be guided in a mostly empty core with an effective modal index close to unity. Indeed, photonic crystals with two- or three-dimensional periodicity can be seen as generalizations of Bragg mirrors [10]. The simple approach that employs reflection and transmission matrices cannot be applied analytically. The aim of using periodicity in two dimensions, in the PCF cladding, is to achieve an omnidirectional bandgap. Bandgaps can exist for all directions of propagation in the plane of periodicity, and propagation of light in any transverse direction can be forbidden. When a bandgap exists regardless of the propagation direction and polarization, the photonic bandgap is complete. Inserting a defect in the middle of the structure permits the existence of a propagating mode in the perturbed crystal. If the propagation constant of this mode coincides with a bandgap in the transverse plane, then the mode will be confined in the locality of the defect, namely the PCF core.

Hollow-core PBG guidance had to wait until the technology could afford larger air-filling fractions essential to achieve a photonic bandgap. The first fiber had a triangular lattice of holes and a relative big hollow core [23]. The main advantage of hollow-core PCF is its capability to guide almost 100% of the optical power in air, leading to very low nonlinearities, high power thresholds, exotic dispersion properties and relatively low propagation loss. An attenuation of 1.2 dB/km at 1620 nm was demonstrated in 2005 [24]. Recently, a hollow-core PCF was reported, which provides a combination of low losses (3.5 dB/km) and wide bandwidth (160 nm), employed to transmit 37×40 Gbps channels at 0.2904 m/ns, i.e., 1.54 ns/km faster than a conventional fiber. This represents the first experimental demonstration of fiber-based wavelength-division multiplexing data transmission at a speed close to (99.7%) that of light in a vacuum [25]. Moreover, it has been demonstrated that by proper design PCF can guide light by both index- and bandgap-guiding simultaneously, in what is termed as hybrid-guiding (HG). The PCF transmission zone is deeply widened and consists of the juxtaposition of bandgap- and index-guiding zones [26]. The properties of such specialty fibers make them good candidates for new applications as low-loss guidance and high power delivery.

The presence of a microstructured cladding with capillaries running along the fiber axis boosts PCF with features unachievable by their conventional counterparts. Apart from providing a means to tailor the PCF properties, the microholes in the PCF cladding provide a natural platform for their infiltration with various sorts of materials, which can significantly enhance their performance or enable new functionalities. Moreover, thanks to the wavelength dispersion of the effective cladding index, the infiltrated material can strongly interact with guided light via evanescent-field effects in a lab-on-a-fiber platform, not achievable in all-solid optical fibers.

Infiltrated PCF have been used in, for instance, supercontinuum generation [27,28,29], guiding atoms through hollow core PCF [30,31,32,33] and even loading Bose–Einstein condensates into the fiber [34,35]. As another example, increasing attention is focused on PCF infiltrated with liquid crystals (LC) in what has been termed as photonic liquid crystal fibers (PLCF), where the high versatility of PCF is combined with the active properties of LC. For instance, following the first demonstration by Larsen et al. [36], PLCF with thermally [37], electrically [38,39], polarization [40] and optically tunable properties [41] have been demonstrated.

This review focuses on infiltrated photonic crystal fibers for sensing applications. Depending on the PCF type, geometry and the infiltrated material, numerous PCF sensors have been thus far demonstrating targeting sensing of temperature, refractive index, electric and magnetic fields, gases, and pressure among others. The structure of this work is based on the type of infiltrated material, highlighting the particular opportunities and challenges associated with each category.

In particular, Section 2 presents experimental protocols that enable PCF infiltration with gaseous, liquid, or solid materials, discussing the particular techniques, which apply in each of the three cases. Section 3 provides a thorough review of infiltrated PCF sensors, divided in subsections according to the infiltrated material: (i) gases, (ii) liquids, (iii) liquid crystalline materials, (iv) solids and (v) metals and metallic nanoparticles in plasmonic PCF configurations. This way, the possibilities and performance metrics provided by each case are presented and the comparative advantages of using infiltrated PCF as to conventional fiber sensors are evidenced. In each subsection, the key properties of the most performing PCF sensors are presented in a table format, allowing for a direct comparison among the various approaches. Section 4 provides an overview of infiltrated PCF sensors, identifies the challenges to scale up and commercialize this novel technology, and draws some concluding remarks.

## 2. Infiltration of Photonic Crystal Fibers

Different materials have dissimilar properties and characteristics, such that when it comes to their infiltration in PCFs, material-specific protocols have to be followed. In the case of solids, the combination of the PCF with, e.g., the common case of metallic wires, can be done during or after the drawing process in a post-fabrication step by infiltration with melted metal or by deposition.

In the case of liquids, the viscosity of the filling material or mixture to is one of the most critical parameters to be considered. The infiltration time depends strongly on the viscosity and on the capillary forces. For instance, the viscous properties are totally different for LC and polymers, depending strongly on temperature and curing. Capillary forces are sometimes sufficiently strong to fill the PCF microcapillaries, whereas in other cases applying pressure becomes necessary using vacuum chambers. Finally, for gases, there is no need for any kind of material-specific infiltration process, although there are techniques that considerably help. Details on the available infiltration techniques for all three cases are provided in the following subsections.

### 2.1. Photonic Crystal Fiber Infiltration with Gases

The identification of chemical species, including gases, is a very important field in sensing applications. Each chemical compound has a particular absorption spectrum, which can be used in order to identify them. The most common measurement technique is the open-path technique. It consists of measuring the absorption of radiation of the sample within a cell of specific length. In the case of PCF, the interaction between light and gas occurs in the evanescent field region. PCF infiltration with gases does not require any kind of material-specific infiltration process. Nevertheless, the main problem of simply letting the gas fill the fiber by itself is the slow diffusion speed. This can lead to long required times for gas filling and venting the fibers (hours in some cases). The diffusion time depends on the type of PCF, as it will be discussed in Section 3.4. For some applications, an acceleration of infiltration times was demonstrated by increasing the pressure and temperature [42]. However, this option is not suitable for practical implementations of sensor applications, where devices have to be exposed to different samples usually at random temperatures and pressures. Furthermore, temperature control requires additional equipment and it is generally not an easy task to precisely control the temperature inside a PCF, which is necessary so as to take into account changes in the gas properties.

**Figure 3:**
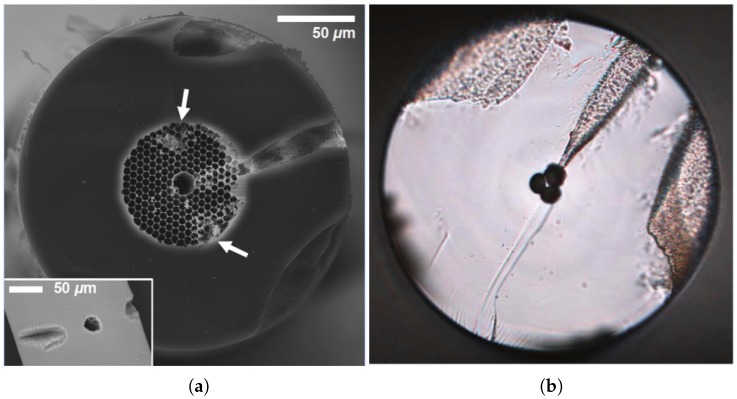
(**a**) SEM image of a micro-channel fabricated in a hollow-core photonic bandgao fiber: arrows indicate damage caused by laser “scoring” (inset shows channel and “scoring” lines on uncoated fiber surface, prior to cleaving); (**b**) optical microscope image showing the cross section of a microchannel fabricated in a SCF, reprinted with permission from [46].

For sensing applications, different approaches for improving the response time have been reported. One simple way is butt-coupling the open end of a PCF to a multi-mode fiber (MMF) using a V-groove [43] fixed into a vacuum chamber [44]. Another option has been reported in [45], using multiple segments of HC-PBF with coupling gaps. Holes were machined into the sides of some glass capillaries and subsequently they were used as couplers between HC-PBGF. Some drawbacks of this approach are high coupling losses and reduced structural stability. Side micromachining on the PCF itself can also be employed for gas infiltration/detection (Figure 3). Although index-guiding in the core is reduced, a sufficient amount of light is guided in lossy modes within the structure. This technique has been demonstrated as one of the best solutions. The main drawback is that the higher the number of holes along the PCF, the greater the fabrication difficulty. This also depends on the PCF type, for example, making holes in index-guiding PCF is considerably easier than in HC-PBF, since the solid core is much more robust than the hollow-core microstructure.

Using the fs-laser micromachining technique, fast-response gas sensors based on multiple micro-channels have been reported (see Section 3.4). Response times in the order of seconds can be achieved. Among all the PCF structures, suspended-core fibers (SCF) offer one of the best solutions, providing high sensitivities and relatively easy channel microfabrication.

### 2.2. Photonic Crystal Fiber Infiltration with Liquids

The first and more intuitive way to fill a PCF with a liquid is by capillarity. One fiber end is dipped into the fluid while the other is left open at room pressure. The necessary condition for the infiltration of the liquid through the holes is the affinity with the PCF material, namely the cohesive forces between the molecules of the liquid must be lower than the adhesion forces of the liquid with the channel material. Extra care is needed in cases where the inner surfaces of the microcapillaries have to be functionalized by coatings or alignment layers, as in liquid-crystal PCF.

Often the selected liquid for infiltration in a PCF can have an excessive viscosity or the time necessary to infiltrate by capillary is too long. In other cases, one end of the PCF is blocked due to splicing to a standard fiber. Under such circumstances, it is necessary to apply an external positive or negative pressure to fill the fiber in. When combined with infiltration in vacuum, these methods become very efficient and enable the infiltration of viscous materials such as liquid crystals or polydimethylsiloxane (PDMS) [47].

When the infiltration of only a certain pattern of microcapillaries is needed, most employed methods rely on the selective blocking of the holes. This can be achieved by directly blocking one by one the selected holes with some other material, such as a polymerizable glue [48,49], or by milling a microchannel into the end facet of a PCF [50]. Another commonly used method is by selectively collapsing the smaller holes with a fusion splicer [51]. The energy density varies with the square of the current density and the temperature of the discharge is proportional to the energy density. The temperature at the midpoint between electrodes falls to a minimum along the electrode’s axis. For this reason, the temperature in the inner cladding is lower and hence the outer cladding holes collapse before. This technique is particularly relevant when only one bigger capillary, e.g., in the central defect core, has to be infiltrated.

**Figure 4:**
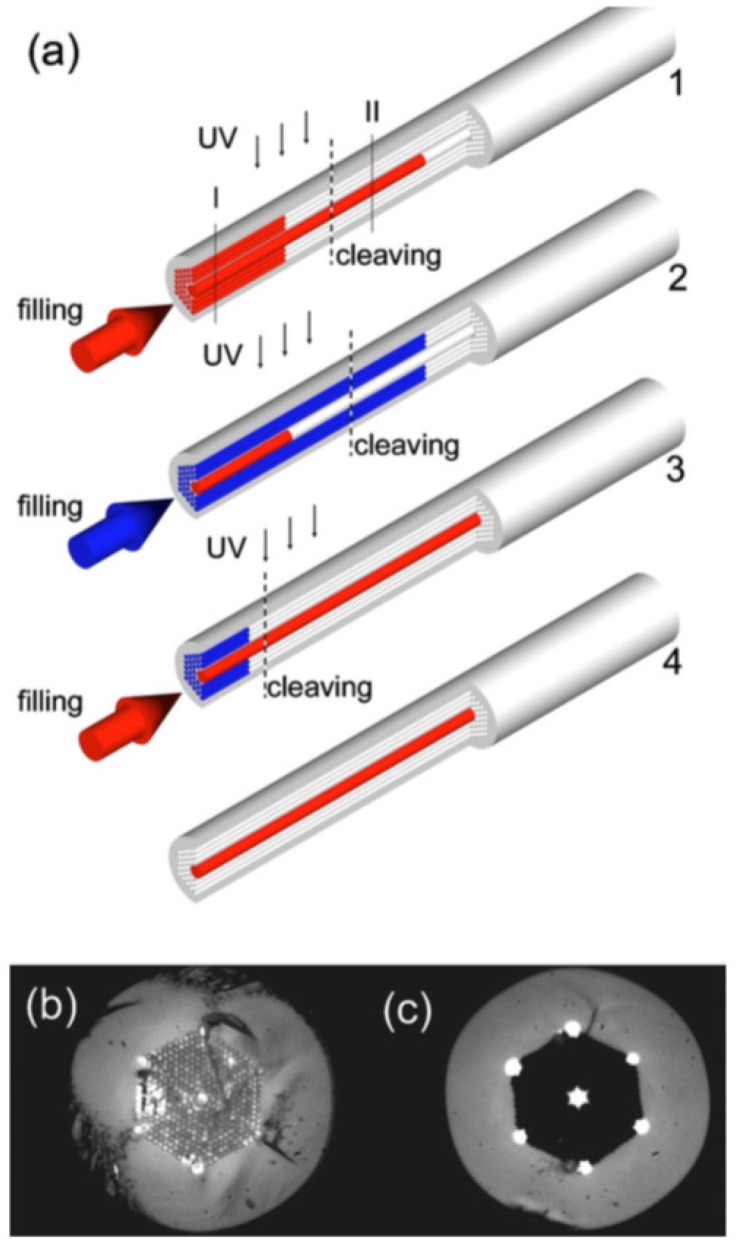
(**a**) Schematic flowchart on selective filling of photonic crystal fibers. The images (**b**,**c**) are optical microscope images of the fiber cross sections at the cleave positions I and II. The light regions correspond to the holes filled with polymer, reprinted with permission from [52].

Another approach takes advantage of the difference in infiltration speed by capillary action among holes with different diameter [52,53]. As the bigger holes are infiltrated faster than the smaller, the difference between the infiltrated lengths for holes of different size increases with time, creating two fronts of infiltrated material within the PCF. By cleaving the fiber at a distance between the two fronts only the bigger holes remain infiltrated with the liquid, as schematically depicted in Figure 4. This technique is simpler, but it is limited by the geometry of the PCF and hence does not allow for arbitrary infiltration patterns.

More advanced methods were also developed, e.g., carving a side-access or using micromachining with a femtosecond laser to expose their inner holes for material infiltration [50,54]. This last method only allows for a limited number of infiltration patterns, contrary to the unrestricted, but more time consuming method of blocking the capillaries one by one.

### 2.3. Photonic Crystal Fiber Infiltration with Solids

**Figure 5:**
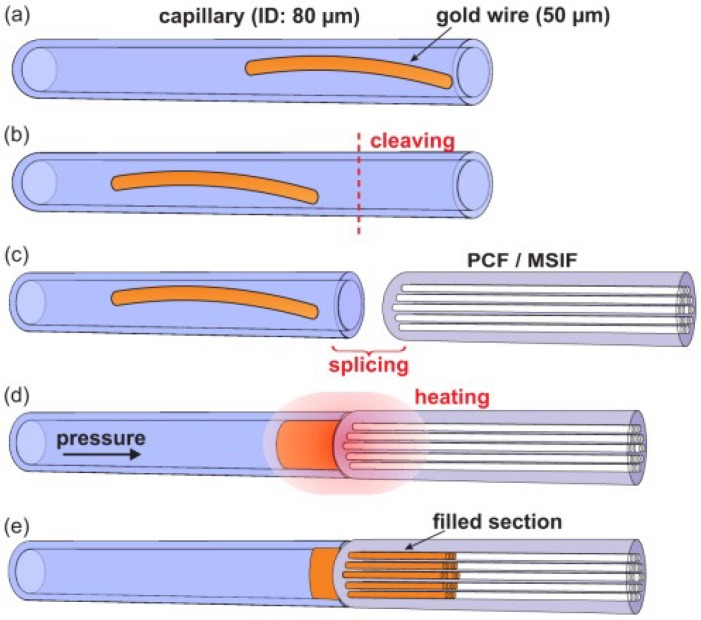
The spliced-fiber pressure-filling technique. (**a**) a gold wire is inserted into a silica capillary; (**b**) the wire is pushed into the capillary using a tungsten wire and the capillary end cleaved off; (**c**) capillary with wire is spliced to a silica fiber with hollow channels; (**d**) the spliced section is heated to the melting point of gold and high pressure argon gas is applied; (**e**) the filled structure. Reprinted with permission from [56].

Different mechanisms have been developed to create hybrid optical fibers with solid materials. PCF infiltration with metals [55,56,57], semiconductors [58] or glasses [59,60] has been thus far reported. Among the different techniques, radio-frequency sputtering, thermal evaporation methods, electroless plating, wet-chemistry, and chemical vapor deposition (CVD) are the most common. The main problem is the surface roughness that appears after fabrication. In addition, some of these methods involve complex organometallic chemistry [61,62]. A technique that overcomes such drawbacks is the pressure-assisted method (Figure 5). It consists of melting the material and introducing it in the holes of the fiber in the liquid state by using high pressure. Successful examples of this technique were demonstrated in [55,57,58]. One of the main difficulties of the pressure-assisted technique is the introduction of the material inside the holes due to the anticapillary force. This effect is considerable in the case of metals that have a high surface tension. As it can be deduced, the bigger the holes are, the smaller the required pressure. An improvement was made in [56] by using fiber splicing at high temperature. Compared to the pressure-cell filling technique, the splice-filling technique is more flexible and safe, requires only small quantities of material, and it is easier to adapt for selective channel filling.

**Figure 6:**
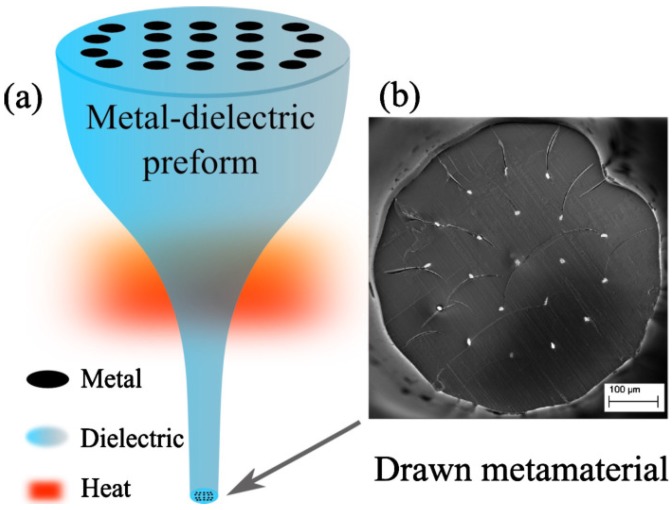
(**a**) schematic of a metal-dielectric preform, drawn into a metamaterial via heating; (**b**) SEM micrograph of a fabricated 590 µm indium-filled PMMA fiber cross-section, reprinted with permission from [65].

Other processes to manufacture hybrid fibers include the codrawing method, which combines the Taylor wire technique [63] with the stack-and-draw procedure, commonly used for silica PCF and poly-methylmethacrylate (PMMA) fibers. The preform is designed by stacking a set of hollow fused silica capillaries around a central silica rod (solid or hollow). Some of these capillaries can been replaced by other material rods as copper [64]. The stack is then slowly introduced in a high temperature furnace while the far end is quickly extracted, as shown in Figure 6. The diameter of the structure is thus controllably reduced and the fiber can be drawn at large lengths without significant variations in its transverse cross-section. In one such example, the material of the extra rods of the preform was indium-filled PMMA, which showed plasmonic response in the THz spectrum [65].

## 3. Sensing Applications

In this section, infiltrated PCF sensors are reviewed based on the type of infiltrated material, namely gases, liquids, liquid crystals, solids, and plasmonic PCF based on metallic layers or nanoparticles. In each subsection, a table summarizes the most relevant results in terms of the sensor type and its key properties. Emphasis is put on the unique sensing opportunities enabled by PCF.

### 3.1. Photonic Crystal Fibers Infiltrated with Gas

The interaction between light and gases is very important in many fields of science. Many particular advantages of PCF, such as enhanced light–matter interaction intensity and length, control over the propagation losses and dispersion properties, and their possibility to host gases, makes them unique for many applications. One of the main differences compared to the case of liquids and solids is that gases can be easily compressed. As a consequence, the nonlinearity and dispersion of the PCF can be tuned by controlling the external pressure. There are several applications of PCF infiltrated with gases, among them some of the most important are pulse delivery [66], power handling [67], self-phase modulation and pulse compression [68], soliton propagation [69], near-infrared and mid-infrared supercontinuum generation [70], quantum optics [71], second harmonic generation [72], four wave mixing [73], and lasing [74]. In the field of sensing, several designs have also been proposed. PCF gas sensors are based on light–gas interaction within the evanescent field region of the PCF. The PCF-sensing mechanism depends on the absorption lines of the corresponding gases. The intensity of light propagating along the PCF can be written as follows [75]:

(1) $$I=e^{-r\alpha lC},$$

where *r* is the relative sensitivity with respect to direct absorption for the same absorber with the same length, α is the absorption coefficient of the gas sample, *l* is the length of the sample and *C* the gas concentration. Sensitivity is usually given as a relative parameter *r*, equal to the ratio between the refractive index of the gas species $n_{g}$ and the effective index of the guided mode $n_{m}$ multiplied by a factor *f* that expresses the fraction of the total evanescent wave power that interacts with the gas [76]. There are two major groups for gas detection based on, first, a special type of microstructured fibers with gas-infiltrated cladding, which resembles index-guiding, solid-core PCF, and, second, bandgap-guiding, hollow-core PCF.

In the first group, the cladding holes reduce the confinement loss and enable light guidance through the core. When the gases are introduced in the holes, the fraction of the evanescent wave is considerably affected. One of the first proposals was made in 2002 by Hoo et al., demonstrating an experimental relative sensitivity of ∼6% [43]. After that, sensitivities of 12.6% and 14.9% at 1.53 and 1.65 µm, namely at the absorption lines of acetylene (C_2_H_2_) and methane (CH_4_) were demonstrated in [77]. The same study reveals that the relative sensitivity increases with the operating wavelength and relative hole size d/Λ, but decreases with the structural size of *d* or Λ for the same ratio d/Λ. By optimizing the relevant parameters, a relative sensitivity of 30% is reported in [78]. The diffusion times in that case were in the order of 1 min for a 7-cm long PCF. By modifying the shape of the holes the sensitivity can be enhanced. A comparison between hexagonal, octagonal and circular air holes in the cladding region is presented in [79]. A random-hole PCF gas sensor was also proposed for the detection of acetylene gas in the optical absorption spectra in [80]. The diffusion times were in the order of tens of minutes.

Another option are SCF characterized by an almost hollow cladding, where the core is supported by a few thin struts connected to the outer cladding and it has a wavelength or sub-wavelength size [81]. This structure, with a small solid core and a large index contrast produces tight confinement of light and strong evanescent field extending into surrounding holes. The coupling to this type of fibers is usually low. In order to solve this problem, a direct coupling scheme was demonstrated between the source and the SCF in [82], by using a vertical-cavity surface-emitting laser (VCSEL), measuring CH_4_ and CO_2_ with a maximum coupling efficiency of 15%. Another option is using an intermediate fiber, showing a total measured loss for the two splices of 0.8 dB [83]. Despite the big holes, the problem of low diffusion rates is still present. In [84], a time of several hours in order to fill completely C_2_H_2_ in a SCF is reported. The main advantage with respect to bandgap-guiding PCF is the ease of making channels to get access to the evanescent field [46]. Even the core can be completely exposed to the external medium [85,86,87] when one of the cladding holes is laterally opened. The exposed-core geometry serves as a versatile platform for real-time evanescent field absorption or fluorescence spectroscopy, with capacity for fast infiltration and quick response to kinetic changes of the analyte [87].

The second group, based on hollow-core PCF, allows for confining both the optical mode and the gas within the central hole. This produces a strong light–gas interaction over distances much longer than other structures used to this end. Since the light–gas interaction is enhanced in the optical power-guiding core, the sensitivity is significantly increased. A typical HC-PBGF operating at 1550 nm has a transmission window of 200 nm and covers the absorption bands of many important gases such as CO, CO_2_, NH_3_, H_2_S, C_2_H_2_, and CH_4_. One of the first examples was demonstrated in 2004, measuring acetylene (C_2_H_2_) [88]. The same year, Ritari et al. demonstrated a high-sensitivity sensor for C_2_H_2_, HCN, CH_4_ and NH_3_ gases in [44]. They also concluded that the use of higher pressure reduces the filling and evacuation time. These effects are studied in detail in [89]. In [90], a big central hole surrounded by a ring of holes was employed. The diffusion times and sensitivity were considerably increased, obtaining a relative sensitivity of 41% at the absorption line of acetylene at 1530.3 nm. The inclusion of channels into the core was reported to improve the gas sensing capabilities [91]. A similar approach was based on introducing a channel in the input of the PCF and closing it with a micromirror [92]. Another example employed femtosecond laser drilling to produce a variable pressure fiber gas cell. Six evenly spaced holes were used over a 2-mm section of a 33-cm long PCF for measuring acetylene. Based on this technique, a fast response PCF methane sensor is proposed in [93]. A diffusion limited response time of ∼3 s and a sensitivity of ∼647 ppm is demonstrated for a 7-cm sensing fiber with seven side-holes separated by 1 cm along the fiber. By modifying the PCF structure, several sensors have been proposed. Two different structures are compared in [94], the results showing a better sensitivity (2.22 times higher) for hexagonal PCF (with six holes in the first ring) compared to an octagonal PCF (with eight holes in the first ring). When the central hole acquires a particular microstructure, different responses are obtained. This technique was introduced in [95], where methylene blue dye was measured with a relative sensitivity of 4.28%. Furthermore, operation in the near-infrared region with considerably improved sensitivity was reported in [96]. In that case, the structure contained five air-hole rings in the cladding and a hexagonal-type microstructure core, and exhibited a relative sensitivity of 42.27%, which is larger than any PCF gas sensor based on interaction with evanescent waves. The confinement loss of the fiber was $4.78\times 10^{-6}$ dB/m. A slotted-core PCF for gas sensing was recently reported in [97]. Numerical results reveal a maximum relative sensitivity of 48.26%. A micro-core PCF-based gas sensor based on elliptical-shaped holes was reported in [98]. According to numerical results, a relative sensitivity of 53.07% can be obtained at 1.33 µm. A different technique employed several segments of HC-PCF to measure methane [45,99], although presenting some drawbacks, mainly the losses between different PCF sections and the difficulty in accurately controlling their junctions.

In addition, several proposals have been made to overcome the limitations of traditional index-guiding and hollow-core PCF. An example are index-guiding PCF with an air core. A relative sensitivity of more than 30% at the methane-relevant wavelength of 1.33 µm was demonstrated in [100]. It was observed that the larger the central hole, the higher the relative sensitivity, although the core had to be small enough to meet the index-guided condition. The sensitivity was found also to increase by reducing the pitch of the PCF lattice. An experimental demonstration of a pure silica defected-core PCF with air core was proposed in [101], showing a relative sensitivity of 4.79% and confinement loss of 32.4 dB/m. A hollow high index ring defect that consists of the central air hole surrounded by a high index GeO_2_ doped SiO_2_ glass ring is introduced in [102]. The proposed PCF provided a relative sensitivity of 5.09%, and a confinement loss of 1.25 dB/m. A similar approach was followed in [103], yielding a relative sensitivity of about 27.58% at the absorption line of methane and hydrogen fluoride, with confinement losses of $1.76\times 10^{-8}$ dB/m. An optimized structure was proposed in [104] by increasing the hole size in the outermost part of the cladding. A relative sensitivity of 32.99% and a confinement loss of $2.59145\times 10^{-5}$ dB/m was reported. A similar approach to [102], but improving the sensitivity, is reported in [105]. By using optimized parameters, a relative sensitivity of 13.23% and a confinement loss to $3.77\times 10^{-6}$ dB/m at the wavelength of 1.33 µm was achieved.

To summarize, PCF are an excellent platform for compact all-fiber gas sensors. The possibility of gas–light interaction over long distances offers high sensitivity in gas detection. One of the main drawbacks is the long diffusion time, which produces slow responses due to time needed for gas filling and venting the fibers. Different approaches for improving the response time have been reported. By using pressure or increased temperature, the filling times are considerably reduced [42], although this solution is not suitable for a practical or commercial device. Introducing side-openings has been demonstrated as an efficient alternative. Still, as the number of channels is increased, the fabrication can become extremely difficult. Moreover, such channels can introduce mode or polarization coupling, thus increasing the noise. Wavelength or phase modulation and advanced digital signal processing may be used to minimize the effect, but more work is needed to prove the effectiveness of such methods [106]. Among all the commented structures, SCFs are the best candidates to achieve high sensitivity and fast response, the fabrication of channels is easier than other configurations and also have the possibility of exposing the core. In addition, both index-guiding PCF and SCF have broad low-loss transmission windows, which makes them suitable for detect multiple gas species. Table 1 provides an overview of the key properties of the most relevant gas-infiltrated PCF sensors.

### 3.2. Photonic Crystal Fibers Infiltrated with Liquids

The microcapillaries in the PCF cladding provide a natural platform for their full or selective infiltration with optical liquids, which extends by far the degrees of freedom in terms of engineering the fiber’s key properties. Optical liquids have demonstrated their capacity of boosting PCF performance in numerous applications, such as in the design of tunable polarizing notch filters [107], nonlinear diffraction and supercontinuum generation [28,108], or dispersion engineering [109,110]. The refractive index (RI) of readily available optical liquids ranges between 1.3 and 2.3 and it can be controlled with very high precision as, e.g., in the commercially available series of refractive index liquids by Cargille-Sacher Laboratories Inc., Cedar Grove (NJ), USA. Such versatility allows for the engineering of both index- and bandgap-guiding PCF with fine-tuned properties, not achievable in solid PCF. Furthermore, the thermo-optic coefficient (TOC) of standard optical liquids is in the range of $-5\times 10^{-4}$ RIU/°C (where RIU stands for refractive index units), namely more than one order of magnitude higher than that of silica, which makes them ideal candidates for the design of tunable fiber components or temperature sensors. Finally, apart from controlling the refractive index of the infiltrated capillaries, liquids can serve as the basis for solutions of nanoparticles or biomolecules, which, combined with the long light–matter interaction lengths, enable advanced functionalities, e.g., sensing of magnetic field or biomolecules.

The performance of PCF sensors is mainly characterized by their sensitivity *S* and detection limit [111]. The former in most cases refers to the wavelength shift of some feature in the PCF transmission spectrum, with respect to the measured quantity, such as temperature, refractive index, strain, or bent radius. Various approaches have been implemented in this respect, including the shifting of the central or edge wavelength of a bandgap or the minima of an interference pattern in multimode/multicore PCF. The detection limit depends on the resolution of the measuring equipment but also on the sharpness of the interrogated spectral feature, for instance, the full-width at half-maximum (FWHM) of a Lorentzian-type resonance. Furthermore, PCF sensors have greatly benefited from already established techniques in standard optical fiber sensors based on the shifting of resonances stemming from fiber Bragg or long-period gratings (LPG) [112,113], which rely on the phase matching and coupling between the fundamental guided mode and counter-propagating or cladding modes, respectively. Various types of grating-based sensors have been consolidated in conventional optical fibers for, e.g., temperature [114,115], strain [115], or humidity [116] measurements. Boosted by advances in optical fiber and material engineering, fiber grating sensors were also demonstrated in polymer optical fibers [117,118] and, finally, PCF [119,120,121,122,123,124,125], using a variety of techniques, such as direct laser writing, electric arc discharge, or by mechanical pressure for LPG [126,127,128].

The dependence of PCF properties on the RI of the infiltrated liquid can be directly exploited in the field of refractometric sensing. By engineering a suitable optofluidic platform [148], the unknown liquid infiltrates the PCF and its RI is extracted by monitoring the variations of the PCF transmission/reflection spectrum or the output power at a single wavelength of operation. A straightforward approach is to fully infiltrate a solid-core photonic bandgap fiber (PBF) and track the position of the bandgaps, provided the RI of the analyte is higher than that of silica, or the fiberglass in general. Using this approach, a high resolution of $10^{-6}$ RIU was demonstrated [149]. However, bandgaps are broad spectral features and their central wavelength and bandedges are usually ill-defined. Instead, a traditional way to introduce sharp resonances in fiber spectra is via the inscription of LPG, which couple the fundamental mode guided in the fiber core with leaky cladding modes at discrete wavelengths that satisfy modal matching conditions. Using CO_2_-inscribed LPG combined with complete PCF infiltration with the measurand a sensitivity of 1500 nm/RIU and resolution of $2\times 10^{-5}$ RIU were demonstrated around the biosensing-relevant RI value of 1.33 [129]. Mechanical LPG, which are simpler to fabricate, were also investigated, yielding a sensitivity of 240 nm/RIU [130]. Coupling to cladding modes can also be achieved by bending the fiber, which transforms the effective RI profile at the fiber cross-section. A very high sensitivity value of 32,400 nm/RIU was recorded in a bent PBF exhibiting an avoided-crossing notch dip in the transmittance spectrum [131].

An alternative way to produce very sharp resonances in liquid-infiltrated PCF (LI-PCF) is to employ dual-core PCF where the directional coupling between the two waveguiding cores strongly depends on the analyte RI. In dual-solid-core PCF, the analyte can selectively occupy the capillaries between the two cores and thus affect the coupling length, thus yielding extremely high sensitivity values [150]. In a complementary approach, the analyte selectively infiltrates only one capillary in the PCF cladding, resulting in a second waveguiding core. By operating close to the cutoff of higher modes of the liquid-core, a value of *S* = 30,100 nm/RIU with a detection limit of $4.6\times 10^{-7}$ RIU was experimentally demonstrated [48]. The performance limits of this sensing configuration were investigated in detail, revealing that in realistic scenarios the detection limit can be as low as $7\times 10^{-8}$, at the expense of longer interaction lengths [151].

Temperature sensing in LI-PCF is essentially based on the same design configurations employed for RI-PCF sensors. The sensor’s RI sensitivity translates into thermal sensitivity via the thermo-optic effect of the infiltrated liquid, hence the need for resonant structures infiltrated with high-TOC optical liquids. In one of the earliest examples, notches in LPG-PBF were thermally tuned at a sensitivity of 1.58 nm/°C [132]. Interferometric measurements in a two-mode solid-core induced by local collapse of the microcapillaries via splicing initially showed lower performance [152,153], but in a later experimental demonstration using a compact fiber length of 0.75 cm a sensitivity value of 1.83 nm/°C was recorded [133].

Better performance was achieved by employing a dual-core PCF scheme, as discussed in the context of refractometric sensors. A fully infiltrated dual-solid core demonstrated a sensitivity of 1.9 nm/°C for a 2-cm-long fiber [154], whereas a directional coupler formed among a solid PCF core and two infiltrated capillaries allowed for a sensitivity of 8.8 nm/°C with very good temporal stability [134]. An enhanced sensitivity of 54.3 nm/°C was demonstrated in Ref. [135] by implementing the concept of the close-to-cutoff coupling to LI capillaries [48,151].

Other approaches include single-wavelength power measurements, which can be very sensitive to temperature variations when the RI of the infiltrated liquid approaches that of silica, leading to the spatial leakage of the fundamental mode and an abrupt rise of its confinement losses. This configuration has intrinsically a short temperature range of operation, but it is characterized by high sensitivity and eliminates the need for spectrum analyzers [136]. PCF temperature sensors have also been demonstrated in more elaborate setups, such as a highly-birefringent, alcohol-infiltrated PCF in loop configuration characterized by a sensitivity of 6.6 nm/°C or a PBF fully-infiltrated with toluene, which was used as both a filter and sensor head in an erbium-doped fiber-amplifier laser with S=1.747 nm/°C [155].

It has been mentioned that a means of increasing the RI/temperature sensitivity of LI-PCF is by bending the fiber and thus inducing light coupling from the core towards cladding modes. Similarly, one can design LI-PCF bend sensors based on the same physical principles, e.g., the bent-sensitive coupling between core mode and a single-capillary in a LI-PCF, which showed a linear sensitivity response of −1.2 nm/m^−1^ for a bend-radius up to 10.7^−1^, albeit with high thermal sensitivity [137]. The latter issue was eliminated in a self-referenced ARROW grapefruit-type microstructured optical fiber (MOF) selectively infiltrated with two different optical liquids in order to minimize thermal and strain cross-talk, while maintaining a high sensitivity value of 4.86 nm/m^−1^ [138]. Furthermore, measurement of not only the bend radius, but the direction of deformation as well, was demonstrated in a commercial PCF (LMA-10) selectively infiltrated with a Cargille optical liquid in such a way that strong geometrical birefringence was induced [156].

By taking advantage of the photoelastic effect in silica PCF, namely the reduction of the silica RI when a strain is axially loaded along the fiber, LI-PCF strain sensors have also been demonstrated. By monitoring the coupling properties between the PCF core and a selectively infiltrated capillary, high sensitivity values of 22 pm/µε were achieved, but suffering from high thermal crosstalk as a result of the highly sensitive coupling [157]. By further optimizing the PCF geometry and material selection both strain and temperature sensitivities can be significantly increased to 701.2 pm/µε and 290 nm/°C, respectively [139]. The thermal crosstalk can be mitigated by employing multi-core LI-PCF for simultaneous strain (S=13.01 pm/µε) and temperature (S=14.72 nm/°C) measurements [140], or highly-birefringent PCF in Sagnac loop interferometric configuration with S=25 pm/µε [141].

In a different context, PCF provide an excellent platform for the design of magnetic field sensors, where the infiltrated liquid is a magnetic fluid (MF), i.e., a stable suspension of ferromagnetic nanoparticles, usually iron oxide (Fe_3_O_4_), in a liquid carrier. In the presence of a magnetic field, the fluid is magnetized and its RI is modified according to a Langevin function, which in turn modulates the PCF optical properties. A sensitivity of 24.2 pm/Oe for a 0.6 mg/mL concentration of Fe_3_O_4_ nanoparticles was demonstrated by exploiting the magnetic-field dependent beating between the two polarizations of the fundamental mode in a highly-birefringent MF-PCF [142]. By using a high-index MF and monitoring the bandgap shift, a linear sensitivity of 1.56 nm/Oe was reported in Ref. [143]. Other approaches comprise the excitation of whispering gallery modes in the outer cladding of an MF-PCF [158] or a self-referenced ARROW-type MOF selectively infiltrated with both MF and ethanol along the lines of [140], which yielded S=81 pm/Oe [144]. Recently, a magnetic-ionic-liquid was used as the MF in a selectively-infiltrated PCF in a compact sensor with an almost linear response in the range from 0 to 440 Oe [145].

Finally, LI-PCF have also demonstrated their potential as biochemical sensors, thanks to the enhanced light–matter interaction they offer and the possibility to functionalize the PCF capillaries and/or infiltrate them with biological solutions. In such an experiment, evanescent wave detection of labeled biomolecules in water was demonstrated by exciting cladding-guided modes which overlapped with the infiltrated capillaries [159]. Higher sensitivity can be achieved by allowing light to be guided in the infiltrated PCF parts, such as in a hollow-core PCF infiltrated with dye solution that featured a resolution in dye concentration of $10^{-10}$ M in fluorescence measurements using a 10-cm PCF [146]. Pushing further the capabilities of LI-PCF in biochemical sensing, the thickness of monolayers of poly-L-lysine and double-stranded DNA was measured in PCF with tunable-LPG, by properly functionalizing the capillary walls of the microstructured cladding [147]. Table 2 provides an overview of the key properties of the most relevant liquid-infiltrated PCF sensors.

### 3.3. Photonic Crystal Fibers Infiltrated with Liquid Crystals

Liquid crystals (LC) are a special class of fluid organic materials that show high intrinsic optical birefringence, stemming from their molecular orientational and/or positional order, which is reminiscent of solid crystalline materials. In addition, their molecular/optical axis reorientates in the presence of applied electric or magnetic fields, which makes them an excellent electro-optic material in a broad range of tunable photonic devices [160,161,162,163,164,165,166,167,168,169,170,171,172,173,174,175,176,177], including numerous examples of LC-PCF for optical switching, tunable filtering and polarization control [178,179,180,181].

LC-PCF infiltration and handling techniques are similar to isotropic LI-PCF, although some extra care is needed in order to ensure the desired LC molecular orientation in the non-biased case, i.e., when there is no applied control field. However, LC offer additional options for sensing, a first example being their use as ultrahigh sensitive temperature sensors. Nematic LC show extremely high TOC of their two indices, particularly the extraordinary one, in the temperature range approaching the clearing point $T_{c}$, namely the transition from anisotropic nematic to the isotropic liquid state. Moreover, via molecular engineering, it is possible to shift the clearing point from room temperature to $T_{c}>100$ °C. By exploiting such physical properties, bandedge shift-based temperature LC-PCF sensors have been demonstrated with sensitivities ranging from 7 nm/°C in one of the early examples [37] to 27 nm/°C for a specially engineered LC for room temperature operation [182] and up to the extreme value of 105 nm/°C, albeit at a very narrow range near the clearing point and with a strong nonlinear response [183]. Linearity and broad temperature range can be restored, at the expense of lower sensitivities, as demonstrated in a thermally-sensitive notch filter in PBG [190]. Apart from bandedge shifting, other approaches have been recently proposed for LC-PCF temperature sensors, such as the use of an interferometric dual-core PCF selectively-infiltrated with the common nematic LC material 5CB (4-cyano-4′-pentylbiphenyl) with sensitivity up to 4.91 nm/°C [184], or a LPG-based design theoretically exhibiting an average sensitivity of 13.79 nm/°C in the range of 27 to 50 °C, which increases up to 438.1 nm/°C near the clearing temperature for the common nematic mixture E7 at $T_{c}=58$ °C [185].

Moreover, owing to their electro-optic response, LC-PCF are prime candidates for compact, all-in-fiber electric field sensors, which minimally disturb the measured field and are immune to electromagnetic interference. Their potential has been demonstrated in various prototypes for polarimetric electric field sensing by optimizing the infiltration length and LC materials. Linear response in compact lengths below 1 mm, sensitivity of 20 dB/kV_rms_/mm and estimated resolution of 0.005 kV_rms_/mm have been reported [186,191]. Operation both in transmission and reflection is possible, using standard commercially available PCF and without the need for selective infiltration [192].

Magnetic field sensing has also been demonstrated using whispering-gallery modes (WGM) in a fiber micro-resonator infiltrated with ferronematic liquid crystal, magnetic field sensitivity up to −6.186 pm/Oe [187]. Other LC-PCF sensing applications include bend sensors based in Bragg gratings inscribed on PCF infiltrated with polymer-dispersed LC showing sensitivity of 0.11 nm/m^−1^ [188] and hydrostatic pressure sensors based on the changes of the capillary diameter and hence bandgap positions and polarization state [189]. Table 3 provides an overview of the key properties of the most relevant liquid-crystal–infiltrated PCF sensors.

### 3.4. Photonic Crystal Fibers Infiltrated with Solids

Infiltrating PCF with solid materials provides an alternative way towards the design and implementation of in-fiber sensors. In many aspects, the properties of solid-infiltrated PCF (SI-PCF) are similar to their LI counterparts, since the PCF capillaries are selectively or fully filled with an isotropic material, as in the most widespread variant of polymer-infiltrated PCF. Nevertheless, SI-PCF can provide wider thermal operation ranges and are easier to handle, e.g., splicing with SCF or empty PCF. On the other hand, they lack some of the versatility of LI-PCF, e.g., the possibility of refractometric sensing in SI-PCF is naturally ruled out, as the PCF capillaries are permanently sealed.

In one of the earliest examples, a grapefruit-type polymer microstructured fiber was selectively infiltrated with a UV curable polymer with a high negative TOC in order to thermally tune the fiber’s birefringence [193]. By inverting the principle of operation, the fiber showed capacity for polarimetric temperature sensing with a sensitivity of 0.15 rad/°C/cm. Higher values of 0.37 rad/°C/cm were reported for a 1.4-cm-long highly-birefringent PCF infiltrated with poly-dimethylsiloxane (PDMS) [194]. PDMS, which has a lower index than silica, was also used to measure temperature by recording the thermally-tunable modal confinement and bending losses of an index-guiding PCF [47]. Being an elastic material, PDMS also shows potential for the design of PCF strain sensors.

Highly-sensitive thermal sensors based on the bandedge-shift effect have been demonstrated by infiltrating PCF with silicone oil [49] and optical adhesives (NOA65) [195], exhibiting absolute sensitivities of 1.35 and 4.034 nm/°C, respectively. In a different approach, the thermo-optic mismatch between two arms of a Mach–Zehnder interferometer formed in a two-core PCF selectively infiltrated with a low-index polymer yielded a sensitivity of 1.595 nm/°C, in terms of the minima dips in the output interferometric spectrum, although requiring a rather large interaction length [196]. A less sensitive (0.18 nm/°C), but very compact (0.8 mm) and having a wide range thermal sensor was demonstrated in a selectively infiltrated PCF with Ge [58]. In general, semiconductors can be infiltrated in PCF at high pressure, provided their melting point is relatively low compared to that of silica, which makes Ge a promising candidate. Using a similar approach, it is also possible to fill PCF with metals, which gives rise to plasmonic effects and novel sensing configurations, which are revised in the dedicated Section 3.5.

More specific applications include in-fiber humidity sensors, based on PCF infiltration with hydroscopic polymer in hydrogel (Agarose), which features a linear change of refractive index over a wide humidity range. By employing an interferometric configuration based on the excitation of core and cladding modes in a PCF via microhole collapsing, a compact (1 mm) fiber sensor head working in reflection was demonstrated [197]. Thanks to its stability, sub-second response, low thermal sensitivity and all-in-fiber approach, this type of humidity sensor can be exploited as a breathing sensor for patients during magnetic resonance imaging [198] or be integrated in larger setups, combined, for instance, with FBG [199], for simultaneous measurement of temperature and humidity. Table 4 provides an overview of the key properties of the most relevant solid-infiltrated PCF sensors.

A particular type of SI-PCF comprises PCF infiltrated with metallic layers/coatings or metallic nanoparticles inside the PCF microcapillaries, which support plasmonic resonances. Given the increased amount of research dedicated in this kind of PCF and their technology, we review the field of plasmonic PCF-based sensors separately in the following subsection.

### 3.5. Plasmonic Photonic Crystal Fibers

**Figure 7:**
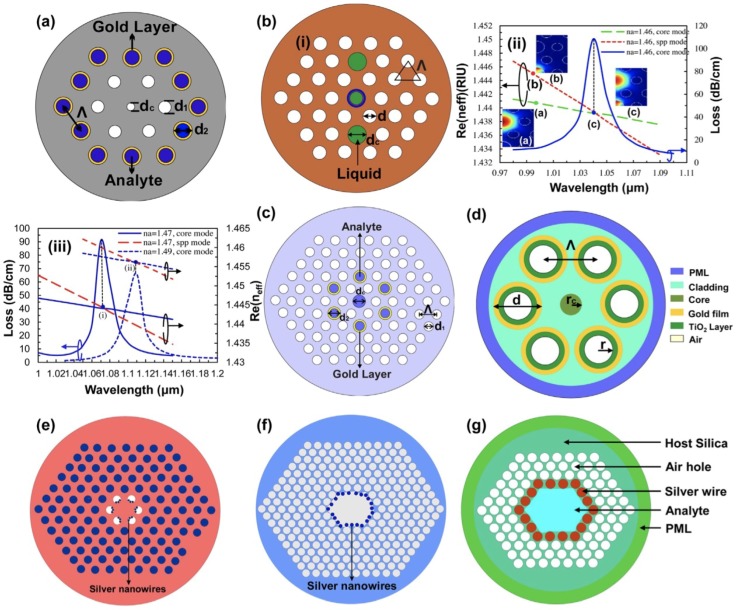
Metal coating-based plasmonic PCF sensors. (**a**) gold coated in the second ring ($d_{c}=0.45\Lambda$, $d_{1}=0.6\Lambda$, $d_{2}=0.8\Lambda$, Λ=2 µm and gold layer thickness equal to 40 nm); (**b**) (**i**) selectively silver deposited core ($d_{c}=0.8\Lambda$, $d_{1}=0.6\Lambda$, $d_{2}=0.8\Lambda$, Λ=2 µm and silver layer thickness equal to 40 nm), (**ii**) field distribution with phase matching phenomena; and (**iii**) phase matching phenomena shifted with varying analyte RI. (**c**) Selectively gold-coated with liquid-filled core ($d_{c}=0.8\Lambda$, $d_{1}=0.5\Lambda$, $d_{2}=0.8\Lambda$, Λ=2 µm and gold layer thickness, t=40 nm); (**d**) multiple holes coated with gold-TiO_2_ layer ($r_{c}=3.5$ µm, r=6 µm, Λ=13 µm, gold layer thickness equal to 30 nm and TiO_2_ layer thickness equal to 75 nm); (**e**) liquid and silver nanowire filled temperature sensor; (**f**) hollow-core filled with liquid and silver nanowires and (**g**) silver-wire filled HC-PBF, reprinted with permission from [200].

Materials that possess a small positive imaginary and a negative real dielectric constant such as noble metals in the visible/infrared (VIS/IR) spectrum are capable of supporting surface plasmon resonances, i.e., coherent oscillations of the surface conduction electrons excited by electromagnetic radiation. In certain cases, such resonances, termed as surface plasmon polaritons (SPP), can be propagating along a metal-dielectric interface, for distances in the order of tens to hundreds micrometers, while decaying evanescently in the plane transverse to the direction of propagation. SPP sensors have attracted much attention in the last few decades thanks to their high sensitivity and broad range of sensing applications. The special characteristics of PCF enable overcoming typical drawbacks of conventional prism and standard optical fiber-based SPP sensors. The great flexibility in the geometry design allows for the control of the core-guided leaky mode propagation. By using different types of structures, the profile of the evanescent field can be optimized. The core-cladding diameter and position can be tuned to obtain single mode propagation, thus increasing the sensitivity and full scale of the sensor. In the case of PCF-SPP sensors, various designs have been proposed in order to simplify the process of introducing metallic layers in the holes, as shown in Figure 7 [200].

Several designs where a plasmonic layer is infiltrated inside the holes have been thus far proposed. These designs aim to improve the detection resolution by increasing the phase matching between the core and plasmonic modes. Figure 7a shows a PCF consisting of two rings of hexagonal holes, where the metallic layer is infiltrated in the second ring, resulting in a metal-coated surface inside the microcapillaries. The central air hole and the presence of the infiltrated fluid in the second ring serve the purpose of phase matching. The interference of the guided modes produces three resonant peaks and the sensor resolution is $3\times 10^{-5}$ RIU [201]. A similar structure, but using graphene and silver as coating layer, is presented in [202]. The graphene layer solves the problem of silver oxidation and increases the adsorption of molecules. A sensitivity of 2520 nm/RIU and a resolution of $3.97\times 10^{-5}$ RIU is demonstrated. Moreover, it has been shown that the use of only one hole for sensing can produce interesting results [203]. In particular, a sensitivity of 9000 nm/RIU between 1.33 and 1.53 is achieved, corresponding to a resolution of $1.11\times 10^{-5}$ RIU.

In Figure 7b, the coating of the central hole and the selectively filled analyte channels induce an intense evanescent field that results in strong coupling between the core-guided mode and the SPP mode [204]. Figure 7b (ii) shows the electric field distribution for an analyte RI of 1.46. The range of the resonant wavelength shift is from 1040 to 1070 nm for a change of analyte RI from 1.46 to 1.47, yielding an RI sensitivity of 3000 nm/RIU [204]. Figure 7b (iii) shows the dispersion relation of the core-guided mode for $n_{a}=1.47$ (solid lines) and $n_{a}=1.49$ (dashed lines). By increasing the refractive index of the analyte, the energy transfer from the core-guided mode to the SPP mode is reduced. The inclusion of the coating layer in the two big holes instead of the center hole was demonstrated in [205]. The average sensitivity was 7040 nm/RIU (gold layer) from 1.40 to 1.42 with high linearity. Another similar deign to Figure 7b, but using six big holes surrounding the core instead of two, was proposed in [206]. The results were sensitives both positive and negative, 3600 nm/RIU for a range of 1.45–1.46 and −5500 nm/RIU for a range of 1.50–1.53, respectively. The use of only the central core was proposed in [207]. A sensitivity of 10,448.5 nm/RIU in the RI range 1.33–1.45 was demonstrated. A similar approach was followed in [208], but combining graphene with silver, as a biosensor with a sensitivity of 10.000 nm/RIU in the range between 1.43 and 1.46 and a high resolution of $10^{-6}$ RIU. The configuration of Figure 7c can also produce both positive and negative sensitivity, by placing the metallic coating in the holes surrounding the core. Thanks to this design, a minimum loss value of 80 dB/cm is obtained for an analyte with RI of 1.485 [209].

Another approach is based on studying the effect of oxides in the coating. In the PCF shown in Figure 7d, the inclusion of a gold-TiO_2_ layer and liquid infiltrated inside the holes improves the sensing performance. A refractive-index resolution of $2.7\times 10^{-5}$ (sensitivity S≃370/RIU) for the liquid analyte and a minimum loss value of 58 dB/cm is demonstrated. Moreover, it can operate at near-infrared wavelengths due to high refractive index of TiO_2_ [210]. A different option exploits the use of indium tin oxide (ITO) [211]. The resonance is around the telecommunication window and it can be tuned by varying the ITO thickness or intrinsic properties. The sensor shows a refractive index sensitivity as high as 2000 nm/RIU and resolution of $5\times 10^{-5}$ RIU. Structures diverging from the classical PCF have also been proposed. A novel diamond ring fiber (DRF)-based sensor for refractive index sensing was designed and fabricated [212], yielding a sensitivity of 6000 nm/RIU in the RI range of 1.33–1.39. The sensing resolution was $1.67\times 10^{-5}$ and $1.97\times 10^{-5}$ RIU by following the wavelength and amplitude interrogation methods, respectively.

One of the main problems of these techniques is the complexity of the fabrication processes. The fabrication process is simplified in [213], by coating two open-ring channels on the outer region of the PCF, where the analyte can penetrate the channels easily. An average spectral sensitivity of 5500 nm/RIU and a maximum sensing resolution of $7.69\times 10^{-6}$ RIU is demonstrated. Other techniques use wires instead of metallic layers. In the case depicted in Figure 7e, silver nanowires are embedded in the first ring of the PCF, which was used to measure temperature with a sensitivity of 2.7 nm/°C [214]. A different example employing nanowires is reported in [215], demonstrating a refractive index sensor with resolution of $5\times 10^{-5}$ RIU and maximum loss of 22 dB/cm, better than in the case of similar structures coated with metal film. A different structure is used in Figure 7f, where the nanowires are embedded in the hollow core of the PCF, resulting in a refractive index sensor with sensitivity of 14,240 nm/RIU [216]. In Figure 7g, the hollow core is infiltrated with the analyte, achieving a maximum sensitivity of 2151 nm/RIU [217]. By using only one wire, a sensitivity of 5500 nm/RIU is demonstrated. More complex structures have also been proposed. A triangular lattice and four large-size channels based on surface plasmon resonance was investigated in [218]. In such a sensor, two gold wires obtained by chemical reaction are filled in two air holes between the upper and lower channels. The results show a sensitivity of 3233 nm/RIU for RI from 1.63 to 1.79. Finally, it was demonstrated in [219] that the maximum sensitivity of the sensor is relatively stable for randomly filled nanowires, which is very convenient in terms of fabrication and application of the sensor.

On the other hand, localized surface plasmon resonances (LSPR) are produced when there is a confinement of a surface plasmon in a particle with size smaller than incident wavelength. As LSPR cannot propagate, the impinging light excites the nanoparticle collective electron oscillations that enhance the near-field amplitude while decaying rapidly with distance from the nanoparticle. The electric field near the surface is enhanced and the optical absorption is maximized at the plasmon resonant frequency. Recent technological achievements, allowing the fabrication of nanoparticles having different sizes and shapes, have boosted the study of the interaction between light and these nanosystems. When the material employed for filling the PCF holes is doped with metallic nanoparticles, LSRP can be generated upon excitation with external light of a given wavelength. Unlike the case of SPP, LSPR can be excited directly without any complex wavevector matching conditions. On the other hand, they are more sensitive to the environment than the SPP.

Gold nanoshells, consisting of silica cores coated with thin gold shells, were infiltrated in a PCF in [228]. Only the first layer of air holes was designed to be the sensing channels by infiltrating them with the gold nanoshells. A sensitivity of 4111.4 nm/RIU and resolution of $2.45\times 10^{-5}$ RIU for 1.33–1.38 were observed. The most common way to manipulate nanoparticles is by means of a host material. In practice, it is difficult to obtain homogeneous samples of dispersed nanoparticles. Common host materials are PDMS [229] or LC [230]. In the case of LC, the most common NP for use with LC are magnetic (Fe_x_O_y_), insulating (ZnO, TiO_2_, Si_3_N_4_), ferroelectric (e.g., BaTiO_3_) and metallic (e.g., plasmonic Au or Ag) nanoparticles, as well as semiconductor quantum dots (CdS, CdSe, CdTe) [231]. For example, a mixture of gold nanoparticles and LC was proposed as a temperature sensor in a polymer optical fiber, with a maximum sensitivity of $64\times 10^{-2}$ dB/°C when metallic NP are used [232]. In addition, the PCF thermo-optical response studied in [233,234] shows a big variation in the properties of transmitted light in small temperature variations. These effects could be used in high-resolution temperature sensors. In [235], a nanoparticle-doped PDMS sample is proposed to be infiltrated in a PCF. It produces a highly birefringent optical response and could be used as temperature sensor with a sensitivity of 0.053 nm/°C. Table 5 provides an overview of the key properties of the most relevant plasmonic PCF sensors.

It is important to note that PCF-SPP sensors have some important considerations. As it was mentioned in Section 2, the inclusion of a metallic layer or wires is experimentally difficult. Placing these materials close to the core causes losses related to the enhancement of the fields at the lossy metallic surfaces. In the case of LSPR sensors, the lack of experimental works demonstrate the extreme difficulty to obtain nanoparticle samples that can be readily infiltrated in PCF. These drawbacks limit the practical realization of this type of sensors. The technology has to be improved considerable in order to have commercial sensors based on plasmonic effects.

## 4. Discussion

A comprehensive review on infiltrated PCF has been presented in the previous sections, highlighting the fundamentals and fabrication of PCF infiltrated with different materials. In the case of solid materials, different mechanisms have been developed to fabricate infiltrated PCF. Among them, RF sputtering, thermal evaporation methods, electroless plating, wet-chemistry, and chemical vapor deposition are the most common. Despite this, pressure-assisted splice-filling has been demonstrated to be the most straightforward and reliable technique. In the case of liquids, the methods to infiltrate the PCF are similar to infiltration of melted metals, with the advantage that temperatures and viscosities are usually easier to handle. For gases, there is no need of any specific mechanism, but high pressure and temperature always helps with the process.

It has been demonstrated how the infiltration of PCF microcapillaries with various types of materials provides new opportunities for practical applications, among which, sensors are the most relevant and with the most commercial potential. The review of infiltrated PCF sensors has been presented in terms of the type of infiltrated material, namely solids, plasmonic coatings and nanoparticles, isotropic liquids, liquid crystals, and gases. The microcapillaries in the PCF cladding provide a natural platform for their full or selective infiltration with optical liquids, which extends by far the degrees of freedom in terms of engineering the fiber’s key properties. Various approaches have been implemented in this respect, including the shifting of the central or edge wavelength of a bandgap, resonances stemming from fiber Bragg gratings or LPG, or the minima of an interference pattern in multimode/multicore PCF. The dependence of PCF properties on the RI of the infiltrated liquid can be directly exploited in the field of refractometric sensing. In addition, temperature sensing in LI-PCF is essentially based on the same design configurations employed for RI-PCF sensors. By taking advantage of the photoelastic effect in silica PCF, namely the reduction of the silica RI when a strain is axially loaded along the fiber, LI-PCF strain sensors have also been demonstrated. In a different context, PCF provides an excellent platform for the design of magnetic field sensors, where the infiltrated liquid is a magnetic fluid. Finally, LI-PCF have also demonstrated their potential as biochemical sensors, thanks to the enhanced light–matter interaction they offer and the possibility to functionalize the PCF capillaries and/or infiltrate them with biological solutions. LC-infiltrated PCF with liquid crystals share some common features with their counterparts infiltrated with liquid isotropic materials, but also provide unique capabilities, such as electric-field sensing via the LC electro-optic effect, much larger TOC for temperature sensing and polarization-dependent interrogation schemes.

Infiltrating PCF with solid materials provides an alternative way towards the design and implementation of in-fiber sensors. Again, in many aspects, the properties of solid-infiltrated PCF are similar to those of liquid-infiltrated PCF, since the capillaries are selectively or fully filled with an isotropic material, as in the most widespread variant of polymer-infiltrated PCF. Nevertheless, SI-PCF can provide wider thermal operation ranges and are easier to handle, e.g., splicing with standard single-mode fibers or empty PCF. On the other hand, they lack some of the versatility of LI-PCF, e.g., the possibility of refractometric sensing in SI-PCF is naturally ruled out, as the PCF capillaries are permanently sealed. Most important applications are highly-sensitive thermal sensors and in-fiber humidity sensors.

When gases are introduced in the PCF holes, the optical response can be used to identify the type of gas. These fibers can be used to make compact all-fiber gas sensors. The possibility of gas–light interaction over long distances produce high sensitivities in gas detection. One of the main drawbacks is the long diffusion time, which produce slow responses due to time taken for gas filling and venting the fibers. Different approaches for improving the response time have been reported. The most promising is the inclusion of side-openings along the PCF. SCFs are the best candidates for achieving high sensitivity and fast responses; the fabrication of channels is easier than other configurations and also has the possibility to expose the core. In addition, both index-guiding PCF and SCF have broad low-loss transmission windows, which make them suitable for detecting multiple gas species. Finally, loading PCF with plasmonic metallic coatings or nanoparticles has also been proposed as a promising way to boost their sensing performance, such as in refractometry, provided the induced damping losses can be efficiently tamed.

Overall, infiltrated PCF emerged as a promising and competitive sensing technology. Their capacity to measure numerous physical quantities, such as temperature, pressure, or strain, detect and to quantify the presence chemical compounds and even biological matter has been demonstrated in numerous experiments. Some techniques are still at their youth, for example, plasmonic PCF, where the majority of this kind of sensors are based on theoretical results. Their fabrication relies on still immature technologies, making it difficult to obtain repetitively the target performance. Despite this, the reported results show a huge potential to improve actual gas and biochemical sensors. Infiltrated IG-PCF and HC-PCF can be considered in a much more mature technological stage, as significant advances in chemical sensing, gas-based nonlinear optics, high power delivery, pulse compression have been demonstrated. Problems such as cross-sensitivity can be addressed, taking advantage of the versatility of PCF as a platform for selective infiltration.

Thanks to such demonstrated capabilities, the research field of infiltrated PCF sensors has grown exponentially in the last years, even if commercial devices are not yet available. Nevertheless, when looking at the number of patents, the perspectives are highly optimistic. For instance, a search in Google’s patent database on infiltrated PCF gas, temperature, strain or pressure sensor returns thousands of results. Although such data serve more as a general indicator, e.g., in many cases, patents overlap as the same PCF sensor can measure several parameters, there is a constant increase in the number of patents every year. This clearly demonstrates the trend and the potential of infiltrated PCF to develop commercial all-fiber sensors in the near future in several key technological fields, such as environmental and biochemical sensing, medical diagnostics, food safety, and security.

## Figures and Tables

**Table 1:** Properties of most relevant gas-infiltrated PCF sensors.

Application	PCF Type	Wavelength (nm)	Sensitivity	Ref.
Gases	IG-PCF	NA	6%	[43]
Acetylene (C_2_H_2_) Methane (CH_4_)	IG-PCF	1530 1650	12.6% 14.9%	[77]
Gases	IG-PCF	750 1750	30%	[78]
Oxygen (O_2_) Methane (CH_4_) Carbon dioxide (CO_2_)	SCF	763 1674 2004	7% 13% 27%	[82]
Acetylene (C_2_H_2_)	HC-PBF	1530	41%	[90]
Methane (CH_4_) Hydrogen fluoride (HF)	HC-PBF	1330	42.27%	[96]
Gases	HC-PBF	NA	48.26%	[97]
Methane (CH_4_)	HC-PBF	1330	53.07%	[98]
Methane (CH_4_)	IG-PCF (air core)	1330	30%	[100]
Methane (CH_4_) Hydrogen fluoride (HF)	IG-PCF (air core)	1330	27.58%	[103]
Gases	IG-PCF (air core)	NA	32.99%	[104]

**Table 2:** Properties of most relevant liquid-infiltrated PCF sensors.

Application	PCF Type	Wavelength (nm)	Sensitivity	Resolution	Ref.
Refractive index	IG-PCF	1050	1500 nm/RIU	$2\times 10^{-5}$	[129]
Refractive index	IG-PCF	1420	240 nm/RIU	$4.1\times 10^{-5}$	[130]
Refractive index	BG-PCF	1350–1550	32,400 nm/RIU	$3.8\times 10^{-6}$	[131]
Refractive index	IG-PCF	1500	30,100 nm/RIU	$4.6\times 10^{-7}$	[48]
Temperature	BG-PCF	1200–1250	1.58 nm/°C	NA	[132]
Temperature	IG-PCF	1250	1.83 nm/°C	NA	[133]
Temperature	IG-PCF	1550	8.8 nm/°C	NA	[134]
Temperature	IG-PCF	1550	54.3 nm/°C	NA	[135]
Temperature	IG-PCF	1550	1 dB/°C	NA	[136]
Bend radius	IG-PCF	1580	−1.2 nm/m^−1^	NA	[137]
Bend radius	IG-PCF	1545	4.86 nm/m^−1^	NA	[138]
Strain	IG-PCF	650–950	701.2 pm/µε	NA	[139]
Strain	IG-PCF	1500	13.01 pm/µε	NA	[140]
Strain	IG-PCF	1520	25 pm/µε	NA	[141]
Magnetic field	IG-PCF	1560	24.2 pm/Oe	NA	[142]
Magnetic field	BG-PCF	1040–1100	1.56 nm/Oe	0.0064 Oe	[143]
Magnetic field	HC-MOF	1535	81 pm/Oe	NA	[144]
Magnetic field	IG-PCF	960	−0.01991 dB/Oe	NA	[145]
Fluorescence	HC-PCF	570	NA	$10^{-10}$ M	[146]
Biolayer thickness	IG-PCF	835	1.4 nm/nm	$10^{-4}$ RIU	[147]

**Table 3:** Properties of most relevant liquid-crystal-infiltrated PCF sensors.

Application	PCF Type	Wavelength (nm)	Sensitivity	Ref.
Temperature	BG-PCF	400–800	7 nm/°C	[37]
Temperature	BG-PCF	1550	27 nm/°C	[182]
Temperature	BG-PCF	1200–1550	105 nm/°C	[183]
Temperature	HG-PCF	1400–1600	4.91 nm/°C	[184]
Temperature	IG-PCF	1400–2600	up to 438.1 nm/°C	[185]
Electric field	IG-PCF	1550	20 dB/kV_rms_/mm	[186]
Magnetic field	IG-PCF	1550	−6.186 pm/Oe	[187]
Bend radius	IG-PCF	1550	0.11 nm/m^−1^	[188]
Hydrostatic pressure	BG-PCF	600–670	0.18 rad/m/MPa	[189]

**Table 4:** Properties of most relevant solid-infiltrated PCF sensors.

Application	PCF Type	Wavelength (nm)	Sensitivity	Ref.
Temperature	IG-MOF	1550	0.15 rad/°C/cm	[193]
Temperature	IG-PCF	633	0.37 rad/°C/cm	[194]
Temperature	BG-PCF	680–740	1.35 nm/°C	[49]
Temperature	BG-PCF	1000–1600	4.034 nm/°C	[195]
Temperature	IG-PCF	1550	1.595 nm/°C	[196]
Temperature	IG-PCF	1390	0.18 nm/°C	[58]
Relative humidity	IG-PCF	1550	0.6 dB/%RH	[197]

**Table 5:** Properties of most relevant plasmonic PCF sensors.

Application	PCF Type	Wavelength (nm)	Sensitivity	Resolution	Ref.
Refractive index	IG-PCF	500 to 1500	873 dB/RIU	$3\times 10^{-5}$ RIU	[201]
Refractive index	IG-PCF	750 to 1750	2520 nm/RIU	$3.97\times 10^{-5}$ RIU	[202]
Refractive index	IG-PCF	500 to 1010	9000 nm/RIU	$1.11\times 10^{-5}$ RIU	[203]
Refractive index	IG-PCF	900 to 1200	10,000 nm/RIU	$10^{-6}$ RIU	[203]
Refractive index	HC-PBF	560 to 610	14,240 nm/RIU	$7.02\times 10^{-5}$ RIU	[216]
Refractive index	HC-PBF	1330	5500 nm/RIU	NA	[98]
Refractive index	SCF	400 to 700	227 nm/RIU	NA	[220]
Refractive index	SCF	1550	8360 rad/RIU	$2.1\times 10^{-6}$ RIU	[221]
Temperature	IG-PCF	400–500	2.7 nm/°C	NA	[214]
Temperature	IG-PCF	800–1100	5 nm/°C	NA	[219]
Temperature	IG-PCF	1550	0.315 dB/°C	NA	[222]
Temperature	HC-PBF	NA	0.72 nm/°C	$1.39\times 10^{-2}$ °C	[223]
Temperature	HC-PBF	550–900	3.08 nm/°C	$1.325\times 10^{-2}$ °C	[224]
Temperature	HC-PBF	NA		$2.8\times 10^{-5}$ RIU	[225]
Pressure	IG-PCF	400–800	32.89 nm/N	NA	[226]
Biolayer thickness	IG-PCF	600–750	2.3 nm/nm	0.044 nm	[227]

## References

[1] Kashyap R., Wyatt R., McKee P.F. (1993). Wavelength flattened saturated erbium amplifier using multiple side-tap Bragg gratings. Electron. Lett..

[2] Shigehara M., Satoh T., Inoue A., Hattori Y. (1996). Optical fiber identification system using fiber Bragg gratings. Opt. Fiber Commun..

[3] Hill K.O., Malo B., Bilodeau F., Thériault S., Johnson D.C., Albert J. (1995). Variable-spectral-response optical waveguide Bragg grating filters for optical signal processing. Opt. Lett..

[4] Hill K.O. (1974). Aperiodic distributed-parameter waveguides for integrated optics. Appl. Opt..

[5] Udd E. (1995). An overview of fiber-optic sensors. Rev. Sci. Instrum..

[6] Zubia J., Arrue J. (2001). Plastic optical fibers: An introduction to their technological processes and applications. Opt. Fiber Technol..

[7] Sano Y., Yoshino T. (2003). Fast optical wavelength interrogator employing arrayed waveguide grating for distributed fiber Bragg grating sensors. J. Lightwave Technol..

[8] Koo K.P., Kersey A.D. (1995). Bragg grating-based laser sensors systems with interferometric interrogation and wavelength division multiplexing. J. Lightwave Technol..

[9] Ashoori R. Time domain multiplexing for a Bragg grating strain measurement sensor network. Proceedings of the 13th International Conference on Optical Fiber Sensors.

[10] Moraleda A.T., Montero D.S., Webb D., García C.V. (2014). A self-referenced optical intensity sensor network using POFBGs for biomedical applications. Sensors.

[11] Montalvo J., Tapetado A., Montero D.S., Vázquez C. WDM-PON preventive optical monitoring system with colourless reflectors. Proceedings of the 2016 Optical Fiber Communications Conference and Exhibition (OFC).

[12] Pinzón P.J., Montero D.S., Tapetado A., Torres J.C., Vázquez C. Dual-wavelength speckle-based SI-POF sensor for frequency detection and localization of remote vibrations. Proceedings of the Sixth European Workshop on Optical Fibre Sensors.

[13] Russell P. (2003). Photonic crystal fibers. Science.

[14] Knight J.C., Birks T.A., Russell P.S.J., Atkin D.M. (1996). All-silica single-mode optical fiber with photonic crystal cladding. Opt. Lett..

[15] Birks T.A., Knight J.C., Russell P.S.J. (1997). Endlessly single-mode photonic crystal fiber. Opt. Lett..

[16] Birks T.A., Knight J.C., Mangan B.J., Russell P.S.J. (2001). Photonic crystal fibres: An endless variety. IEICE Trans. Electron..

[17] Knight J.C., Birks T.A., Cregan R.F., Russell P.S.J., de Sandro J.P. (1998). Large mode area photonic crystal fibre. Electron. Lett..

[18] Ortigosa-Blanch A., Knight J.C., Wadsworth W.J., Arriaga J., Mangan B.J., Birks T.A., Russell P.S.J. (2000). Highly birefringent photonic crystal fibers. Opt. Lett..

[19] Dakin J.P., Brown R.G.W. (2006). Handbook of Optoelectronics (Two-Volume Set).

[20] Zolla F., Renversez G., Nicolet A., Kuhlmey B., Guenneau S., Felbacq D., Argyros A., Leon-Saval S. (2011). Foundations of Photonic Crystal Fibres.

[21] Mogilevtsev D., Birks T., Russell P. (1999). Localized function method for modeling defect modes in 2-D photonic crystals. J. Lightwave Technol..

[22] Ferrando A., Silvestre E., Miret J.J., Andrés P., Andrés M.V. (1999). Full-vector analysis of a realistic photonic crystal fiber. Opt. Lett..

[23] Cregan R.F. (1999). Single-mode photonic band gap guidance of light in air. Science.

[24] Roberts P.J., Couny F., Sabert H., Mangan B.J., Williams D.P., Farr L., Mason M.W., Tomlinson A., Birks T.A., Knight J.C. (2005). Ultimate low loss of hollow-core photonic crystal fibres. Opt. Express.

[25] Poletti F., Wheeler N.V., Petrovich M.N., Baddela N., Fokoua E.N., Hayes J.R., Gray D.R., Li Z., Slavík R., Richardson D.J. (2013). Towards high-capacity fibre-optic communications at the speed of light in vacuum. Nat. Photonics.

[26] Perrin M., Quiquempois Y., Bouwmans G., Douay M. (2007). Coexistence of total internal reflexion and bandgap modes in solid core photonic bandgap fibre with intersticial air holes. Opt. Express.

[27] Ranka J.K., Windeler R.S., Stentz A.J. Efficient visible continuum generation in air-silica microstructure optical fibers with anomalous dispersion at 800 nm. Proceedings of the IEEE Conference on Lasers and Electro-Optics.

[28] Zhang R., Teipel J., Giessen H. (2006). Theoretical design of a liquid-core photonic crystal fiber for supercontinuum generation. Opt. Express.

[29] Bozolan A., de Matos C.J., Cordeiro C.M.B., dos Santos E.M., Travers J. (2008). Supercontinuum generation in a water-core photonic crystal fiber. Opt. Express.

[30] Renn M.J., Montgomery D., Vdovin O., Anderson D.Z., Wieman C.E., Cornell E.A. (1995). Laser-guided atoms in hollow-core optical fibers. Phys. Rev. Lett..

[31] Ito H., Nakata T., Sakaki K., Ohtsu M., Lee K.I., Jhe W. (1996). Laser apectroscopy of atoms guided by evanescent waves in micron-sized hollow optical fibers. Phys. Rev. Lett..

[32] Dall R.G., Hoogerland M.D., Baldwin K.G.H., Buckman S.J. (1999). Guiding of metastable helium atoms through hollow optical fibres. J. Opt. B Quant. Semiclass. Opt..

[33] Müller D., Cornell E.A., Anderson D.Z., Abraham E.R.I. (2000). Guiding laser-cooled atoms in hollow-core fibers. Phys. Rev. A.

[34] Christensen C.A., Will S., Saba M., Jo G.B., Shin Y.I., Ketterle W., Pritchard D. (2008). Trapping of ultracold atoms in a hollow-core photonic crystal fiber. Phys. Rev. A.

[35] Krauss T.F. (2008). Why do we need slow light?. Nat. Photonics.

[36] Larsen T., Bjarklev A., Hermann D., Broeng J. (2003). Optical devices based on liquid crystal photonic bandgap fibres. Opt. Express.

[37] Wolinski T.R., Szaniawska K., Ertman S., Lesiak P., Domanski A.W., Dabrowski R., Nowinowski-Kruszelnicki E., Wojcik J. (2006). Influence of temperature and electrical fields on propagation properties of photonic liquid-crystal fibres. Meas. Sci. Technol..

[38] Du F., Lu Y.Q., Wu S.T. (2004). Electrically tunable liquid-crystal photonic crystal fiber. Appl. Phys. Lett..

[39] Haakestad M.W., Alkeskjold T.T., Nielsen M.D., Scolari L., Riishede J., Engan H.E., Bjarklev A. (2005). Electrically tunable photonic bandgap guidance in a liquid-crystal-filled photonic crystal fiber. IEEE Photonics Technol. Lett..

[40] Woliński T.R., Szaniawska K., Bondarczuk K., Lesiak P., Domański A.W., Dąbrowski R., Nowinowski-Kruszelnicki E., Wójcik J. (2005). Propagation properties of photonic crystal fibers filled with nematic liquid crystals. Opto-Electron. Rev..

[41] Alkeskjold T.T., Lægsgaard J., Bjarklev A., Hermann D.S., Anawati A., Broeng J., Li J., Wu S.T. (2004). All-optical modulation in dye-doped nematic liquid crystal photonic bandgap fibers. Opt. Express.

[42] Wynne R.M., Barabadi B., Creedon K.J., Ortega A. (2009). Sub-minute response time of a hollow-core photonic bandgap fiber gas sensor. J. Lightwave Technol..

[43] Hoo Y.L. (2002). Evanescent-wave gas sensing using microstructure fiber. Opt. Eng..

[44] Ritari T., Tuominen J., Ludvigsen H., Petersen J.C., Sørensen T., Hansen T.P., Simonsen H.R. (2004). Gas sensing using air-guiding photonic bandgap fibers. Opt. Express.

[45] Parry J.P., Griffiths B.C., Gayraud N., McNaghten E.D., Parkes A.M., MacPherson W.N., Hand D.P. (2009). Towards practical gas sensing with micro-structured fibres. Meas. Sci. Technol..

[46] Van Brakel A., Grivas C., Petrovich M.N., Richardson D.J. (2007). Micro-channels machined in microstructured optical fibers by femtosecond laser. Opt. Express.

[47] Markos C., Vlachos K., Kakarantzas G. (2010). Bending loss and thermo-optic effect of a hybrid PDMS/silica photonic crystal fiber. Opt. Express.

[48] Wu D.K.C., Kuhlmey B.T., Eggleton B.J. (2009). Ultrasensitive photonic crystal fiber refractive index sensor. Opt. Lett..

[49] Mileńko K., Rutkowska K.A., Woliński T.R. (2013). Numerical and experimental analysis of photonic crystal fiber selectively infiltrated with silicon oil. Acta Phys. Pol. A.

[50] Wang F., Yuan W., Hansen O., Bang O. (2011). Selective filling of photonic crystal fibers using focused ion beam milled microchannels. Opt. Express.

[51] Xiao L., Jin W., Demokan M.S., Ho H.L., Hoo Y.L., Zhao C. (2005). Fabrication of selective injection microstructured optical fibers with a conventional fusion splicer. Opt. Express.

[52] Huang Y., Xu Y., Yariv A. (2004). Fabrication of functional microstructured optical fibers through a selective-filling technique. Appl. Phys. Lett..

[53] Nielsen K., Noordegraaf D., Sørensen T., Bjarklev A., Hansen T.P. (2005). Selective filling of photonic crystal fibres. J. Opt. A Pure Appl. Opt..

[54] Martelli C., Olivero P., Canning J., Groothoff N., Gibson B., Huntington S. (2007). Micromachining structured optical fibers using focused ion beam milling. Opt. Lett..

[55] Lee H.W., Schmidt M.A., Tyagi H.K., Sempere L.P., Russell P.S.J. (2008). Polarization-dependent coupling to plasmon modes on submicron gold wire in photonic crystal fiber. Appl. Phys. Lett..

[56] Lee H.W., Schmidt M.A., Russell R.F., Joly N.Y., Tyagi H.K., Uebel P., Russell P.S.J. (2011). Pressure-assisted melt-filling and optical characterization of Au nano-wires in microstructured fibers. Opt. Express.

[57] Schmidt M.A., Sempere L.N.P., Tyagi H.K., Poulton C.G., Russell P.S.J. (2008). Waveguiding and plasmon resonances in two-dimensional photonic lattices of gold and silver nanowires. Phys. Rev. B.

[58] Tyagi H.K., Schmidt M.A., Sempere L.P., Russell P.S. (2008). Optical properties of photonic crystal fiber with integral micron-sized Ge wire. Opt. Express.

[59] Markos C., Yannopoulos S.N., Vlachos K. (2012). Chalcogenide glass layers in silica photonic crystal fibers. Opt. Express.

[60] Schmidt M.A., Granzow N., Da N., Peng M., Wondraczek L., Russell P.S.J. (2009). All-solid bandgap guiding in tellurite-filled silica photonic crystal fibers. Opt. Lett..

[61] Takeyasu N., Tanaka T., Kawata S. (2005). Metal deposition deep into microstructure by electroless plating. Jpn. J. Appl. Phys..

[62] Sazio P.J.A. (2006). Microstructured optical fibers as high-pressure microfluidic reactors. Science.

[63] Donald I.W., Metcalfe B.L. (1996). The preparation, properties and applications of some glass-coated metal filaments prepared by the Taylor-wire process. J. Mater. Sci..

[64] Hou J., Bird D., George A., Maier S., Kuhlmey B., Knight J.C. (2008). Metallic mode confinement in microstructured fibres. Opt. Express.

[65] Tuniz A., Kuhlmey B.T., Lwin R., Wang A., Anthony J., Leonhardt R., Fleming S.C. (2010). Drawn metamaterials with plasmonic response at terahertz frequencies. Appl. Phys. Lett..

[66] Michieletto M., Lyngsø J.K., Jakobsen C., Lægsgaard J., Bang O., Alkeskjold T.T. (2016). Hollow-core fibers for high power pulse delivery. Opt. Express.

[67] Hädrich S., Rothhardt J., Demmler S., Tschernajew M., Hoffmann A., Krebs M., Liem A., de Vries O., Plötner M., Fabian S. (2016). Scalability of components for kW-level average power few-cycle lasers. Appl. Opt..

[68] Heckl O.H., Saraceno C.J., Baer C.R.E., Südmeyer T., Wang Y.Y., Cheng Y., Benabid F., Keller U. (2011). Temporal pulse compression in a xenon-filled Kagome-type hollow-core photonic crystal fiber at high average power. Opt. Express.

[69] Ouzounov D.G. (2003). Generation of megawatt optical solitons in hollow-core photonic band-gap fibers. Science.

[70] Cassataro M., Novoa D., Günendi M.C., Edavalath N.N., Frosz M.H., Travers J.C., Russell P.S.J. (2017). Generation of broadband mid-IR and UV light in gas-filled single-ring hollow-core PCF. Opt. Express.

[71] Finger M.A., Iskhakov T.S., Joly N.Y., Chekhova M.V., Russell P.S.J. (2015). Raman-free, noble-gas-filled photonic-crystal fiber source for ultrafast, very bright twin-beam squeezed vacuum. Phys. Rev. Lett..

[72] Ménard J.M., Köttig F., Russell P.S.J. (2016). Broadband electric-field-induced LP_01 and LP_02 second harmonic generation in Xe-filled hollow-core PCF. Opt. Lett..

[73] Konorov S.O., Fedotov A.B., Zheltikov A.M. (2003). Enhanced four-wave mixing in a hollow-core photonic-crystal fiber. Opt. Lett..

[74] Jones A.M., Nampoothiri A.V.V., Ratanavis A., Fiedler T., Wheeler N.V., Couny F., Kadel R., Benabid F., Washburn B.R., Corwin K.L. (2011). Mid-infrared gas filled photonic crystal fiber laser based on population inversion. Opt. Express.

[75] Ruddy V. (1990). An effective attenuation coefficient for evanescent wave spectroscopy using multimode fiber. Fiber Integr. Opt..

[76] Stewart G., Norris J., Clark D.F., Culshaw B. (1991). Evanescent-wave chemical sensors: A theoretical evaluation. Int. J. Optoelectron..

[77] Hoo Y.L., Jin W., Shi C., Ho H.L., Wang D.N., Ruan S.C. (2003). Design and modeling of a photonic crystal fiber gas sensor. Appl. Opt..

[78] Ho H.L., Hoo Y.L., Jin W., Ju J., Wang D.N., Windeler R.S., Li Q. (2007). Optimizing microstructured optical fibers for evanescent wave gas sensing. Sens. Actuators B.

[79] Asaduzzaman S., Ahmed K., Arif M.F.H., Morshed M. Application of microarray-core based modified photonic crystal fiber in chemical sensing. Proceedings of the 2015 International Conference on Electrical & Electronic Engineering.

[80] Pickrell G., Peng W., Wang A. (2004). Random-hole optical fiber evanescent-wave gas sensing. Opt. Lett..

[81] Kaiser P., Astle H.W. (1974). Low-loss single-material fibers made from pure fused silica. Bell Syst. Tech. J..

[82] Chen J., Hangauer A., Strzoda R., Euser T.G., Chen J.S.Y., Scharrer M., Russell P.S.J., Amann M.C. Sensitivity limits for near-infrared gas sensing with suspended-core PCFs directly coupled with VCSELs. Proceedings of the Conference on Lasers and Electro-Optics.

[83] Dong L., Thomas B.K., Fu L. (2008). Highly nonlinear silica suspended core fibers. Opt. Express.

[84] Webb A.S. (2007). Suspended-core holey fiber for evanescent-field sensing. Opt. Eng..

[85] Cox F.M., Lwin R., Large M.C.J., Cordeiro C.M.B. (2007). Opening up optical fibres. Opt. Express.

[86] Warren-Smith S.C., Ebendorff-Heidepriem H., Foo T.C., Moore R., Davis C., Monro T.M. (2009). Exposed-core microstructured optical fibers for real-time fluorescence sensing. Opt. Express.

[87] Kostecki R., Ebendorff-Heidepriem H., Davis C., McAdam G., Warren-Smith S.C., Monro T.M. (2012). Silica exposed-core microstructured optical fibers. Opt. Mater. Express.

[88] Hoo Y.L., Jin W., Ho H.L., Ju J., Wang D.N. (2005). Gas diffusion measurement using hollow-core photonic bandgap fiber. Sens. Actuators B.

[89] Henningsen J., Hald J. (2008). Dynamics of gas flow in hollow core photonic bandgap fibers. Appl. Opt..

[90] Kassani S.H., Khazaeinezhad R., Jung Y., Kobelke J., Oh K. (2015). Suspended Ring-Core Photonic Crystal Fiber Gas Sensor With High Sensitivity and Fast Response. IEEE Photonics J..

[91] Li X., Pawłat J., Liang J., Xu G., Ueda T. (2009). Fabrication of photonic bandgap fiber gas cell using focused ion beam cutting. Jpn. J. Appl. Phys..

[92] Li X., Liang J., Oigawa H., Ueda T. (2011). Doubled optical path length for photonic bandgap fiber gas cell using micromirror. Jpn. J. Appl. Phys..

[93] Jin W., Qi L.F., Ho H.L., Cao Y.C. Gas detection with micro and nano-engineered optical fibers. Proceedings of the Conference on Optical Sensors 2012, Micro and Nano-Engineered Sensors.

[94] Das S., Jayaraman V. (2014). SnO2: A comprehensive review on structures and gas sensors. Prog. Mater. Sci..

[95] Cordeiro C.M.B., Franco M.A.R., Chesini G., Barretto E.C.S., Lwin R., Cruz C.H.B., Large M.C.J. (2006). Microstructured-core optical fibre for evanescent sensing applications. Opt. Express.

[96] Morshed M., Hassan M.I., Roy T.K., Uddin M.S., Razzak S.M.A. (2015). Microstructure core photonic crystal fiber for gas sensing applications. Appl. Opt..

[97] Asaduzzaman S., Ahmed K., Paul B.K., Liu T., Jiang S., Landgraf R. (2016). Slotted-core photonic crystal fiber in gas-sensing application. Advanced Sensor Systems and Applications VII.

[98] Asaduzzaman S., Ahmed K. (2016). Proposal of a gas sensor with high sensitivity, birefringence and nonlinearity for air pollution monitoring. Sens. Bio-Sens. Res..

[99] Carvalho J.P., Lehmann H., Bartelt H., Magalhães F., Amezcua-Correa R., Santos J.L., Van Roosbroeck J., Araújo F.M., Ferreira L.A., Knight J.C. (2009). Remote system for detection of low-levels of methane based on photonic crystal fibres and wavelength modulation spectroscopy. J. Sens..

[100] Zhang Z., Zhang F., Zhang M., Ye P. (2008). Gas sensing properties of index-guided PCF with air-core. Opt. Laser Technol..

[101] Yu X., Sun Y., Ren G.B., Shum P., Ngo N.Q., Kwok Y.C. (2008). Evanescent field absorption sensor using a pure-silica defected-core photonic crystal fiber. IEEE Photonic Technol. Lett..

[102] Park J., Lee S., Kim S., Oh K. (2011). Enhancement of chemical sensing capability in a photonic crystal fiber with a hollow high index ring defect at the center. Opt. Express.

[103] Morshed M., Hasan M.I., Razzak S.M.A. (2015). Enhancement of the sensitivity of gas sensor based on microstructure optical fiber. Photonic Sens..

[104] Olyaee S., Naraghi A. (2013). Design and optimization of index-guiding photonic crystal fiber gas sensor. Photonic Sens..

[105] Olyaee S., Naraghi A., Ahmadi V. (2014). High sensitivity evanescent-field gas sensor based on modified photonic crystal fiber for gas condensate and air pollution monitoring. Optik Int. J. Light Electron Opt..

[106] Jin W., Ho H.L., Cao Y.C., Ju J., Qi L.F. (2013). Gas detection with micro- and nano-engineered optical fibers. Opt. Fiber Technol..

[107] Zografopoulos D.C., Kriezis E.E. (2007). Tunable optical fiber polarization elements based on long-period gratings inscribed in birefringent microstructured fibers. J. Opt. Soc. Am. B.

[108] Rosberg C.R., Bennet F.H., Neshev D.N., Rasmussen P.D., Bang O., Krolikowski W., Bjarklev A., Kivshar Y.S. (2007). Tunable diffraction and self-defocusing in liquid-filled photonic crystal fibers. Opt. Express.

[109] Gundu K.M., Kolesik M., Moloney J.V., Lee K.S. (2006). Ultra-flattened-dispersion selectively liquid-filled photonic crystal fibers. Opt. Express.

[110] Zografopoulos D.C., Vázquez C., Kriezis E.E., Yioultsis T.V. (2011). Dual-core photonic crystal fibers for tunable polarization mode dispersion compensation. Opt. Express.

[111] Kuhlmey B.T., Eggleton B.J., Wu D.K.C. (2009). Fluid-filled solid-core photonic bandgap fibers. J. Lightwave Technol..

[112] Kersey A.D., Davis M.A., Patrick H.J., LeBlanc M., Koo K.P., Askins C.G., Putnam M.A., Friebele E.J. (1997). Fiber grating sensors. J. Lightwave Technol..

[113] Bhatia V., Vengsarkar A.M. (1996). Optical fiber long-period grating sensors. Opt. Lett..

[114] Djambova T.V., Mizunami T. (2000). Simultaneous sensing of temperature and displacement using a multimode fiber Bragg grating. Jpn. J. Appl. Phys..

[115] Lim J., Yang Q., Jones B.E., Jackson P.R. (2002). Strain and temperature sensors using multimode optical fiber Bragg gratings and correlation signal processing. IEEE Trans. Instrum. Meas..

[116] Kronenberg P., Rastogi P.K., Giaccari P., Limberger H.G. (2002). Relative humidity sensor with optical fiber Bragg gratings. Opt. Lett..

[117] Yuan W., Stefani A., Bache M., Jacobsen T., Rose B., Herholdt-Rasmussen N., Nielsen F.K., Andresen S., Sørensen O.B., Hansen K.S. (2011). Improved thermal and strain performance of annealed polymer optical fiber Bragg gratings. Opt. Commun..

[118] Woyessa G., Fasano A., Stefani A., Markos C., Nielsen K., Rasmussen H.K., Bang O. (2016). Single mode step-index polymer optical fiber for humidity insensitive high temperature fiber Bragg grating sensors. Opt. Express.

[119] Frazão O., Santos J.L., Araújo F.M., Ferreira L.A. (2008). Optical sensing with photonic crystal fibers. Laser Photonics Rev..

[120] Martelli C., Canning J., Groothoff N., Lyytikainen K. (2005). Strain and temperature characterization of photonic crystal fiber Bragg gratings. Opt. Lett..

[121] Chen G., Liu L., Jia H., Yu J., Xu L., Wang W. (2003). Simultaneous pressure and temperature measurement using Hi-Bi fiber Bragg gratings. Opt. Commun..

[122] Geernaert T., Luyckx G., Voet E., Nasilowski T., Chah K., Becker M., Bartelt H., Urbanczyk W., Wojcik J., Waele W.D. (2009). Transversal load sensing with fiber Bragg gratings in microstructured optical fibers. IEEE Photonics Technol. Lett..

[123] Bhatia V., Campbell D., Claus R.O., Vengsarkar A.M. (1997). Simultaneous strain and temperature measurement with long-period gratings. Opt. Lett..

[124] Venugopalan T., Sun T., Grattan K.T.V. (2008). Long period grating-based humidity sensor for potential structural health monitoring. Sens. Actuators A.

[125] Ju J., Jin W. (2011). Long period gratings in photonic crystal fibers. Photonic Sens..

[126] Chiavaioli F., Baldini F., Tombelli S., Trono C., Giannetti A. (2017). Biosensing with optical fiber gratings. Nanophotonics.

[127] Jeong Y., Yang B., Lee B., Seo H.S., Choi S., Oh K. (2000). Electrically controllable long-period liquid crystal fiber gratings. IEEE Photonics Technol. Lett..

[128] Noordegraaf D., Scolari L., Lægsgaard J., Rindorf L., Alkeskjold T.T. (2007). Electrically and mechanically induced long period gratings in liquid crystal photonic bandgap fibers. Opt. Express.

[129] Rindorf L., Bang O. (2008). Highly sensitive refractometer with a photonic-crystal-fiber long-period grating. Opt. Lett..

[130] Yu X., Shum P., Ren G.B. (2008). Highly sensitive photonic crystal fiber-based refractive index sensing using mechanical long-period grating. IEEE Photonics Technol. Lett..

[131] Han T., Liu Y.G., Wang Z., Zou B., Tai B., Liu B. (2010). Avoided-crossing-based ultrasensitive photonic crystal fiber refractive index sensor. Opt. Lett..

[132] Steinvurzel P., Moore E.D., Mägi E.C., Eggleton B.J. (2006). Tuning properties of long period gratings in photonic bandgap fibers. Opt. Lett..

[133] Geng Y., Li X., Tan X., Deng Y., Hong X. (2014). Compact and ultrasensitive temperature sensor with a fully liquid-filled photonic crystal fiber Mach–Zehnder interferometer. IEEE Sens. J..

[134] Yang M., Wang D.N. (2012). Photonic crystal fiber with two infiltrated air holes for temperature sensing with excellent temporal stability. J. Lightwave Technol..

[135] Wang Y., Yang M., Wang D.N., Liao C.R. (2011). Selectively infiltrated photonic crystal fiber with ultrahigh temperature sensitivity. IEEE Photonics Technol. Lett..

[136] Wang R., Yao J., Miao Y., Lu Y., Xu D., Luan N., Musideke M., Duan L., Hao C. (2013). A reflective photonic crystal fiber temperature sensor probe based on infiltration with liquid mixtures. Sensors.

[137] Chen H.F., Wang Y., Wang D.N. (2015). Selectively infiltrated PCF for directional bend sensing with large bending range. IEEE Photonics Technol. Lett..

[138] Gao R., Lu D., Cheng J., Meiqi Z. (2017). Self-referenced antiresonant reflecting guidance mechanism for directional bending sensing with low temperature and strain crosstalk. Opt. Express.

[139] Luo M., Liu Y.G., Wang Z., Han T., Wu Z., Guo J., Huang W. (2013). Twin-resonance-coupling and high sensitivity sensing characteristics of a selectively fluid-filled microstructured optical fiber. Opt. Express.

[140] Lin C., Wang Y., Huang Y., Liao C., Bai Z., Hou M., Li Z., Wang Y. (2017). Liquid modified photonic crystal fiber for simultaneous temperature and strain measurement. Photonics Res..

[141] Han T., Liu Y.G., Wang Z., Guo J., Yu J. (2018). A high sensitivity strain sensor based on the zero-group- birefringence effect in a selective-filling high birefringent photonic crystal fiber. IEEE Photonics J..

[142] Thakur H.V., Nalawade S.M., Gupta S., Kitture R., Kale S.N. (2011). Photonic crystal fiber injected with Fe_3_O_4_ nanofluid for magnetic field detection. Appl. Phys. Lett..

[143] Zu P., Chan C.C., Gong T., Jin Y., Wong W.C., Dong X. (2012). Magneto-optical fiber sensor based on bandgap effect of photonic crystal fiber infiltrated with magnetic fluid. Appl. Phys. Lett..

[144] Gao R., Lu D.F., Cheng J., Jiang Y., mei Qi Z. (2017). Temperature-compensated fibre optic magnetic field sensor based on a self-referenced anti-resonant reflecting optical waveguide. Appl. Phys. Lett..

[145] Liang H., Liu Y., Li H., Han S., Zhang H., Wu Y., Wang Z. (2018). Magnetic-ionic-liquid-functionalized photonic crystal fiber for magnetic field detection. IEEE Photonics Technol. Lett..

[146] Smolka S., Barth M., Benson O. (2007). Highly efficient fluorescence sensing with hollow core photonic crystal fibers. Opt. Express.

[147] Rindorf L., Jensen J.B., Dufva M., Pedersen L.H., Høiby P.E., Bang O. (2006). Photonic crystal fiber long-period gratings for biochemical sensing. Opt. Express.

[148] Yong D., Ng W.L., Yu X., Chan C.C. (2013). A compact opto-fluidic platform for chemical sensing with photonic crystal fibers. Sens. Actuators A.

[149] Yu X., Shum P., Ren G.B., Ngo N.Q. (2008). Photonic crystal fibers with high index infiltrations for refractive index sensing. Opt. Commun..

[150] Town G.E., Yuan W., McCosker R., Bang O. (2010). Microstructured optical fiber refractive index sensor. Opt. Lett..

[151] Wu D.K.C., Lee K.J., Pureur V., Kuhlmey B.T. (2013). Performance of refractive index sensors based on directional couplers in photonic crystal fibers. J. Lightwave Technol..

[152] Qiu S.J., Chen Y., Xu F., Lu Y.Q. (2012). Temperature sensor based on an isopropanol-sealed photonic crystal fiber in-line interferometer with enhanced refractive index sensitivity. Opt. Lett..

[153] Wang S., Liu Y.G., Wang Z., Han T., Xu W., Wang Y., Wang S. (2013). Intermodal interferometer based on a fluid-filled two-mode photonic crystal fiber for sensing applications. Appl. Opt..

[154] Du J., Liu Y., Wang Z., Liu Z., Zou B., Jin L., Liu B., Kai G., Dong X. (2008). Thermally tunable dual-core photonic bandgap fiber based on the infusion of a temperature-responsive liquid. Opt. Express.

[155] Yang X., Lu Y., Liu B., Yao J. (2017). Fiber ring laser temperature sensor based on liquid-filled photonic crystal fiber. IEEE Sens. J..

[156] Wang Y., Tan X., Jin W., Liu S., Ying D., Hoo Y.L. (2010). Improved bending property of half-filled photonic crystal fiber. Opt. Express.

[157] Wang Y., Liao C.R., Wang D.N. (2012). Embedded coupler based on selectively infiltrated photonic crystal fiber for strain measurement. Opt. Lett..

[158] Mahmood A., Kavungal V., Ahmed S.S., Farrell G., Semenova Y. (2015). Magnetic-field sensor based on whispering-gallery modes in a photonic crystal fiber infiltrated with magnetic fluid. Opt. Lett..

[159] Jensen J.B., Pedersen L.H., Hoiby P.E., Nielsen L.B., Hansen T.P., Folkenberg J.R., Riishede J., Noordegraaf D., Nielsen K., Carlsen A. (2004). Photonic crystal fiber based evanescent-wave sensor for detection of biomolecules in aqueous solutions. Opt. Lett..

[160] D’Alessandro A., Asquini R., Bellini R.P., Donisi D., Beccherelli R. Integrated optic devices using liquid crystals: Design and fabrication issues. Proceedings of the 49th SPIE Annual Meeting and International Symposium on Optical Science and Technology.

[161] Bellini B., Larchanché J.F., Vilcot J.P., Decoster D., Beccherelli R., d’Alessandro A. (2005). Photonic devices based on preferential etching. Appl. Opt..

[162] D’Alessandro A., Donisi B.D., Beccherelli R., Asquini R. (2006). Nematic liquid crystal optical channel waveguides on silicon. IEEE J. Quant. Electron..

[163] Bellini B., d’Alessandro A., Beccherelli R. (2007). A method for butt-coupling optical fibres to liquid crystal planar waveguides. Opt. Mater..

[164] D’Alessandro A., Donisi D., Sio L.D., Beccherelli R., Asquini R., Caputo R., Umeton C. (2008). Tunable integrated optical filter made of a glass ion-exchanged waveguide and an electro-optic composite holographic grating. Opt. Express.

[165] Gilardi G., Sio L.D., Beccherelli R., Asquini R., d’Alessandro A., Umeton C. (2011). Observation of tunable optical filtering in photosensitive composite structures containing liquid crystals. Opt. Lett..

[166] Infusino M., Ferraro A., Luca A.D., Caputo R., Umeton C. (2012). POLYCRYPS visible curing for spatial light modulator based holography. J. Opt. Soc. Am. B.

[167] Zografopoulos D.C., Asquini R., Kriezis E.E., d’Alessandro A., Beccherelli R. (2012). Guided-wave liquid-crystal photonics. Lab Chip.

[168] Zografopoulos D.C., Beccherelli R. (2012). Plasmonic variable optical attenuator based on liquid-crystal tunable stripe waveguides. Plasmonics.

[169] Zografopoulos D.C., Beccherelli R. (2013). Liquid-crystal-tunable metal–insulator–metal plasmonic waveguides and Bragg resonators. J. Opt..

[170] Zografopoulos D.C., Beccherelli R. (2013). Design of a vertically coupled liquid-crystal long-range plasmonic optical switch. Appl. Phys. Lett..

[171] Algorri J.F., Urruchi V., Bennis N., Sánchez-Pena J.M. (2014). Modal liquid crystal microaxicon array. Opt. Lett..

[172] Algorri J.F., del Pozo V.U., Sanchez-Pena J.M., Oton J.M. (2014). An autostereoscopic device for mobile applications based on a liquid crystal microlens array and an OLED display. J. Disp. Technol..

[173] Algorri J.F., Lallana P.C., Urruchi V., Sanchez-Pena J.M. (2015). Liquid crystal temperature sensor based on three electrodes and a high-resistivity layer. IEEE Sens. J..

[174] Algorri J.F., Urruchi V., Bennis N., Morawiak P., Sanchez-Pena J.M., Oton J.M. (2016). Integral imaging capture system with tunable field of view based on liquid crystal microlenses. IEEE Photonics Technol. Lett..

[175] Algorri J.F., Bennis N., Herman J., Kula P., Urruchi V., Sánchez-Pena J.M. (2017). Low aberration and fast switching microlenses based on a novel liquid crystal mixture. Opt. Express.

[176] Algorri J.F., Urruchi V., Bennis N., Morawiak P., Sánchez-Pena J.M., Otón J.M. (2017). Liquid crystal spherical microlens array with high fill factor and optical power. Opt. Express.

[177] Algorri J.F., Bennis N., Urruchi V., Morawiak P., Sánchez-Pena J.M., Jaroszewicz L.R. (2017). Tunable liquid crystal multifocal microlens array. Sci. Rep..

[178] Zografopoulos D.C., Pitilakis A., Kriezis E.E. (2015). Liquid crystal-infiltrated photonic crystal fibres for switching applications. Optofluidics, Sensors and Actuators in Microstructured Optical Fibers.

[179] Zografopoulos D.C., Kriezis E.E. (2009). Tunable polarization properties of hybrid-guiding liquid-crystal photonic crystal fibers. J. Lightwave Technol..

[180] Pitilakis A.K., Zografopoulos D.C., Kriezis E.E. (2011). In-line polarization controller based on liquid-crystal photonic crystal fibers. J. Lightwave Technol..

[181] Zografopoulos D.C., Pitilakis A.K., Kriezis E.E. (2013). Dual-band electro-optic polarization switch based on dual-core liquid-crystal photonic crystal fibers. Appl. Opt..

[182] Alkeskjold T.T., Lægsgaard J., Bjarklev A., Hermann D.S., Broeng J., Li J., Gauza S., Wu S.T. (2006). Highly tunable large-core single-mode liquid-crystal photonic bandgap fiber. Appl. Opt..

[183] Sun B., Huang Y., Wang C., He J., Liao C., Yin G., Zhou J., Liu S., Zhao J., Wang Y. Ultra-sensitive temperature sensor based on liquid crystal infiltrated photonic crystal fibers. Proceedings of the Fifth Asia-Pacific Optical Sensors Conference.

[184] Xu Z., Li B., Hu D.J.J., Wu Z., Ertman S., Wolinski T., Tong W., Shum P.P. (2018). Hybrid photonic crystal fiber for highly sensitive temperature measurement. J. Opt..

[185] Du C., Wang Q., Zhao Y. (2018). Electrically tunable long period gratings temperature sensor based on liquid crystal infiltrated photonic crystal fibers. Sens. Actuators A.

[186] Mathews S., Farrell G., Semenova Y. (2011). All-fiber polarimetric electric field sensing using liquid crystal infiltrated photonic crystal fibers. Sens. Actuators A.

[187] Mahmood A., Kavungal V., Ahmed S.S., Kopcansky P., Zavisova V., Farrell G., Semenova Y. (2017). Magnetic field sensing using whispering-gallery modes in a cylindrical microresonator infiltrated with ferronematic liquid crystal. Opt. Express.

[188] Zito G., Pissadakis S. (2013). Holographic polymer-dispersed liquid crystal Bragg grating integrated inside a solid core photonic crystal fiber. Opt. Lett..

[189] Wolinski T.R., Czapla A., Ertman S., Tefelska M., Domanski A.W., Wojcik J., Nowinowski-Kruszelnicki E., Dabrowski R. (2008). Photonic liquid crystal fibers for sensing applications. IEEE Trans. Instrum. Meas..

[190] Noordegraaf D., Scolari L., Lægsgaard J., Alkeskjold T.T., Tartarini G., Borelli E., Bassi P., Li J., Wu S.T. (2008). Avoided-crossing-based liquid-crystal photonic-bandgap notch filter. Opt. Lett..

[191] Mathews S., Farrell G., Semenova Y. (2011). Directional electric field sensitivity of a liquid crystal infiltrated photonic crystal fiber. IEEE Photonics Technol. Lett..

[192] Mathews S., Farrell G., Semenova Y. (2011). Liquid crystal infiltrated photonic crystal fibers for electric field intensity measurements. Appl. Opt..

[193] Kerbage C., Steinvurzel P., Reyes P., Westbrook P.S., Windeler R.S., Hale A., Eggleton B.J. (2002). Highly tunable birefringent microstructured optical fiber. Opt. Lett..

[194] Markos C., Vlachos K., Kakarantzas G. (2011). Guiding and birefringent properties of a hybrid PDMS/silica photonic crystal fiber. SPIE.

[195] Sun B., Wei W., Wang C., Liao C., Xu J., Wan H., Zhang L., Zhang Z., Wang Y. (2016). Solid optical fiber with tunable bandgaps based on curable polymer infiltrated photonic crystal fiber. J. Lightwave Technol..

[196] Naeem K., Kim B.H., Kim B., Chung Y. (2015). High-sensitivity temperature sensor based on a selectively-polymer- filled two-core photonic crystal fiber in-line interferometer. IEEE Sens. J..

[197] Mathew J., Semenova Y., Farrell G. (2012). Relative humidity sensor based on an agarose-infiltrated photonic crystal fiber interferometer. IEEE J. Sel. Top. Quantum Electron..

[198] Mathew J., Semenova Y., Farrell G. (2012). A miniature optical breathing sensor. Biomed. Opt. Express.

[199] Mathew J., Semenova Y., Farrell G. (2013). Fiber optic hybrid device for simultaneous measurement of humidity and temperature. IEEE Sens. J..

[200] Rifat A.A., Ahmed R., Yetisen A.K., Butt H., Sabouri A., Mahdiraji G.A., Yun S.H., Adikan F.R.M. (2017). Photonic crystal fiber based plasmonic sensors. Sens. Actuators B.

[201] Hassani A., Skorobogatiy M. (2006). Design of the microstructured optical fiber-based surface plasmon resonance sensors with enhanced microfluidics. Opt. Express.

[202] Yang X., Lu Y., Liu B., Yao J. (2016). Analysis of graphene-based photonic crystal fiber sensor using birefringence and surface plasmon resonance. Plasmonics.

[203] Wang F., Sun Z., Liu C., Sun T., Chu P.K. (2018). A high-sensitivity photonic crystal fiber (PCF) based on the surface plasmon resonance (SPR) biosensor for detection of density alteration in non-physiological cells (DANCE). Opto-Electron. Rev..

[204] Rifat A., Mahdiraji G., Chow D., Shee Y., Ahmed R., Adikan F. (2015). Photonic crystal fiber-based surface plasmon resonance sensor with selective analyte channels and graphene-silver deposited core. Sensors.

[205] Fan Z., Li S., Liu Q., An G., Chen H., Li J., Chao D., Li H., Zi J., Tian W. (2015). High sensitivity of refractive index sensor based on analyte-filled photonic crystal fiber with surface plasmon resonance. IEEE Photonics J..

[206] Shuai B., Xia L., Liu D. (2012). Coexistence of positive and negative refractive index sensitivity in the liquid-core photonic crystal fiber based plasmonic sensor. Opt. Express.

[207] Qin W., Li S.G., Xue J.R., Xin X.J., Zhang L. (2013). Numerical analysis of a photonic crystal fiber based on two polarized modes for biosensing applications. Chin. Phys. B.

[208] Wang F., Sun Z., Liu C., Sun T., Chu P.K. (2016). A highly sensitive dual-core photonic crystal fiber based on a surface plasmon resonance biosensor with silver-graphene layer. Plasmonics.

[209] Qin W., Li S., Yao Y., Xin X., Xue J. (2014). Analyte-filled core self-calibration microstructured optical fiber based plasmonic sensor for detecting high refractive index aqueous analyte. Opt. Lasers Eng..

[210] Gao D., Guan C., Wen Y., Zhong X., Yuan L. (2014). Multi-hole fiber based surface plasmon resonance sensor operated at near-infrared wavelengths. Opt. Commun..

[211] Dash J.N., Jha R. (2014). SPR biosensor based on polymer PCF coated with conducting metal oxide. IEEE Photonics Technol. Lett..

[212] Ng W.L., Rifat A.A., Wong W.R., Mahdiraji G.A., Adikan F.R.M. (2017). A novel diamond ring fiber-based surface plasmon resonance sensor. Plasmonics.

[213] Liu C., Yang L., Lu X., Liu Q., Wang F., Lv J., Sun T., Mu H., Chu P.K. (2017). Mid-infrared surface plasmon resonance sensor based on photonic crystal fibers. Opt. Express.

[214] Lu Y., Wang M.T., Hao C.J., Zhao Z.Q., Yao J.Q. (2014). Temperature sensing using photonic crystal fiber filled with silver nanowires and liquid. IEEE Photonics J..

[215] Fu X., Lu Y., Huang X., Hao C., Wu B., Yao J. (2011). Surface plasmon resonance sensor based on photonic crystal fiber filled with silver nanowires. Opt. Appl..

[216] Lu Y., Yao J., Yang X., Wang M. (2015). Surface plasmon resonance sensor based on hollow-core PCFs filled with silver nanowires. Electron. Lett..

[217] Biswas T., Chattopadhyay R., Bhadra S.K. (2014). Plasmonic hollow-core photonic band gap fiber for efficient sensing of biofluids. J. Opt..

[218] An G., Li S., Yan X., Zhang X., Yuan Z., Wang H., Zhang Y., Hao X., Shao Y., Han Z. (2016). Extra-broad photonic crystal fiber refractive index sensor based on surface plasmon resonance. Plasmonics.

[219] Luan N., Wang R., Lv W., Lu Y., Yao J. (2014). Surface plasmon resonance temperature sensor based on photonic crystal fibers randomly filled with silver nanowires. Sensors.

[220] Schroder K., Csaki A., Schwuchow A., Jahn F., Strelau K., Latka I., Henkel T., Malsch D., Schuster K., Weber K. (2012). Functionalization of microstructured optical fibers by internal nanoparticle mono-layers for plasmonic biosensor applications. IEEE Sens. J..

[221] Wang G., Liu J., Zheng Z., Yang Y., Xiao J., Li S., Bian Y. (2012). Fluidic sensor based on the side-opened and suspended dual-core fiber. Appl. Opt..

[222] Yu Y., Li X., Hong X., Deng Y., Song K., Geng Y., Wei H., Tong W. (2010). Some features of the photonic crystal fiber temperature sensor with liquid ethanol filling. Opt. Express.

[223] Peng Y., Hou J., Huang Z., Lu Q. (2012). Temperature sensor based on surface plasmon resonance within selectively coated photonic crystal fiber. Appl. Opt..

[224] Liu C., Wang F., Lv J., Sun T., Liu Q., Fu C., Mu H., Chu P.K. (2016). A highly temperature-sensitive photonic crystal fiber based on surface plasmon resonance. Opt. Commun..

[225] Bing P.B., Li Z.Y., Yao J.Q., Lu Y., Di Z.G. (2012). A photonic crystal fiber based on surface plasmon resonance temperature sensor with liquid core. Mod. Phys. Lett. B.

[226] Zhang S., Yu X., Shum P., Zhang Y., Ho H.P., Liu D. (2011). Highly sensitive pressure-induced plasmon resonance birefringence in a silver-coated photonic crystal fiber. J. Phys. Conf. Ser..

[227] Hassani A., Skorobogatiy M. (2009). Photonic crystal fiber-based plasmonic sensors for the detection of biolayer thickness. J. Opt. Soc. Am. B.

[228] Liu B., Lu Y., Yang X., Yao J. (2017). Tunable surface plasmon resonance sensor based on photonic crystal fiber filled with gold nanoshells. Plasmonics.

[229] Algorri J.F., Poudereux D., García-Cámara B., Urruchi V., Sánchez-Pena J.M., Vergaz R., Caño-García M., Quintana X., Geday M.A., Otón J.M. (2016). Metal nanoparticles-PDMS nanocomposites for tunable optical filters and sensors. Opt. Data Process. Storage.

[230] Budaszewski D., Siarkowska A., Chychłowski M., Jankiewicz B., Bartosewicz B., Dąbrowski R., Woliński T.R. (2018). Nanoparticles-enhanced photonic liquid crystal fibers. J. Mol. Liq..

[231] Woliński T.R., Siarkowska A., Budaszewski D., Chychłowski M., Czapla A., Ertman S., Lesiak P., Rutkowska K.A., Orzechowski K., Sala-Tefelska M. (2017). Recent advances in liquid-crystal fiber optics and photonics. Proc. SPIE.

[232] Algorri J.F., Garcia-Camara B., Garcia-Garcia A., Urruchi V., Sanchez-Pena J.M. (2015). Fiber optic temperature sensor based on amplitude modulation of metallic and semiconductor nanoparticles in a liquid crystal mixture. J. Lightwave Technol..

[233] Siarkowska A., Chychłowski M., Woliński T.R., Dybko A. (2016). Titanium nanoparticles doping of 5CB infiltrated microstructured optical fibers. Photonics Lett. Pol..

[234] Siarkowska A., Chychłowski M., Budaszewski D., Jankiewicz B., Bartosewicz B., Woliński T.R. (2017). Thermo- and electro-optical properties of photonic liquid crystal fibers doped with gold nanoparticles. Beilstein J. Nanotechnol..

[235] Poudereux D., Caño-García M., Algorri J.F., García-Cámara B., Sánchez-Pena J.M., Quintana X., Geday M.A., Otón J.M. (2015). Thermally tunable polarization by nanoparticle plasmonic resonance in photonic crystal fibers. Opt. Express.

